# Supplementary material for: Contractile Defect Caused by Mutation in MYBPC3 Revealed under Conditions Optimized for Human PSC-Cardiomyocyte Function
Source: Cell Rep. 2015 Oct 17;13(4):733–45. doi: 10.1016/j.celrep.2015.09.025 (PMC4644234; doi:10.1016/j.celrep.2015.09.025)
Supplement: Document S1. Supplemental Experimental Procedures, Figures S1–S5, and Table S1 [file mmc1.pdf]

Cell Reports

Supplemental Information

Contractile Defect Caused by Mutation in *MYBPC3* Revealed under Conditions Optimized for Human PSC-Cardiomyocyte Function

Matthew J. Birket, Marcelo C. Ribeiro, Georgios Kosmidis, Dorien Ward, Ana Rita Leitoguinho, Vera van de Pol, Cheryl Dambrot, Harsha D. Devalla, Richard P. Davis, Pier G. Mastroberardino, Douwe E. Atsma, Robert Passier, and Christine L. Mummery

Inventory of Supplementary Information for:

"Contractile defect caused by mutation in *MYBPC3* revealed under conditions optimized for human PSC-cardiomyocyte function"

Correspondence to: Christine L Mummery PhD, Leiden University Medical Center, 2300 RC Leiden, The Netherlands. Telephone: +31-71-526 9307 Fax: +31-71-526 8289 Email: c.l.mummery@lumc.nl

- **Figure S1**
- **Figure S2**
- **Figure S3**
- **Figure S4**
- **Figure S5**
- **Table S1.**
- **Supplemental experimental procedures**

Movie S1. Typical spontaneously contracting hESC-cardiomyocytes on micropatterned polyacrylamide in control medium or TID-containing medium. Related to Figure 4.

Movie S2. Typical spontaneously contracting hiPSC-cardiomyocytes, control or HCM, on micropatterned polyacrylamide in TID-containing medium. Related to Figure 5.

Supplementary figure 1

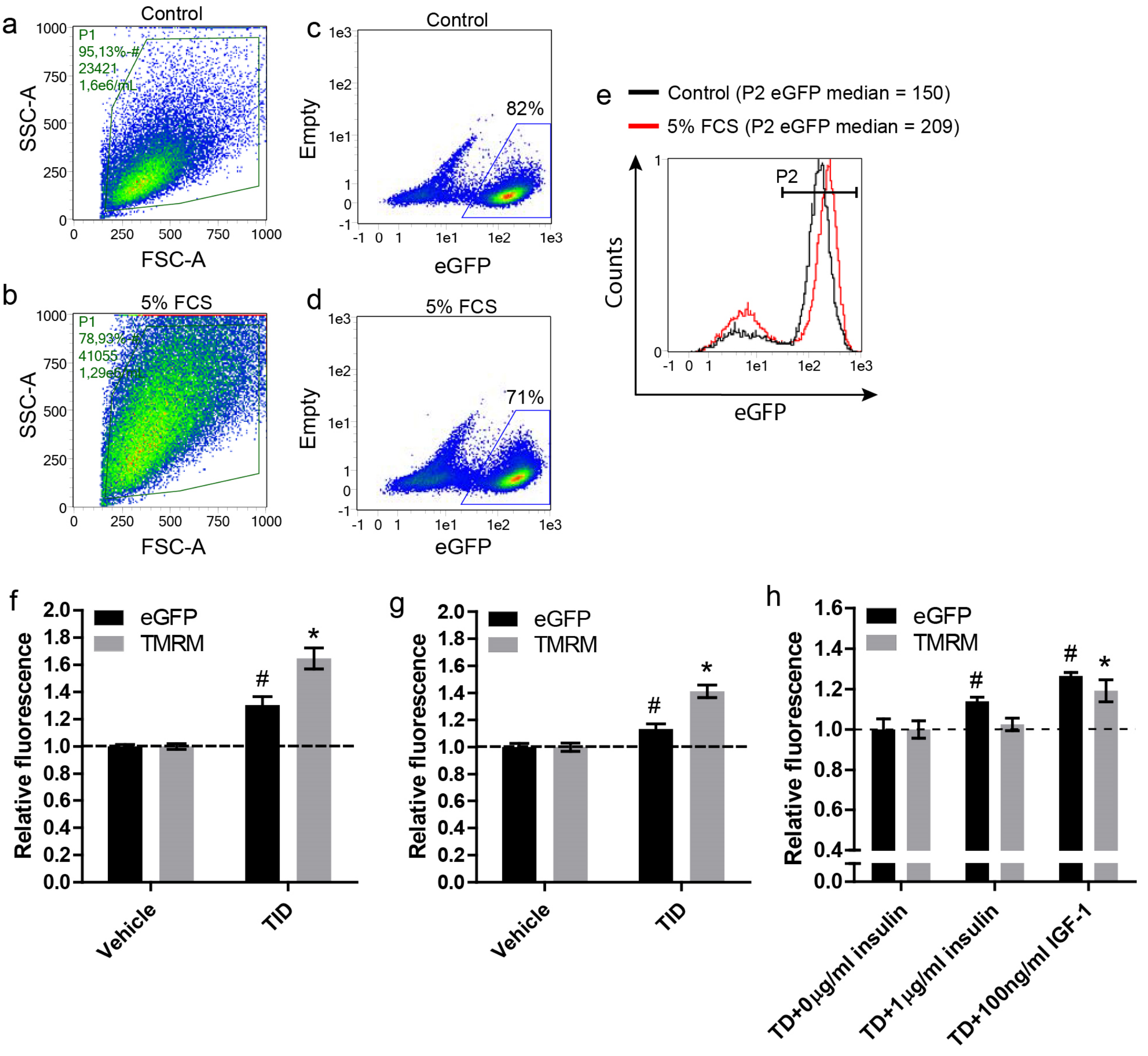

Figure S1. GFP and TMRM measurements in hPSC-derived cardiomyocytes maintained in different conditions. Related to Figure 1. FACS measurement after cardiac differentiation of *NKX2-5*$^{eGFP/w}$ hESCs and maintenance for 5 days under control conditions or in the presence of 5% FCS. (**a**) Scatter plots and P1 gating of control and (**b**) 5% FCS treated cells, (**c**) eGFP fluorescence of the P1 population of control and (**d**) FCS treated cells, (**e**) eGFP fluorescence histogram showing an overlay of the two P1 populations. The median eGFP fluorescence value within the P2 gate is shown above the histogram. (**f**) Relative TMRM and eGFP fluorescence values of M1 *NKX2-5*$^{eGFP/w}$ hESC-derived cardiomyocytes and (**g**) *NKX2-5*$^{eGFP/w}$ hiPSC-derived cardiomyocytes after 5 days of treatment with vehicle-only or TID medium. (**h**) Relative TMRM and eGFP fluorescence of cardiomyocytes after 5 days of treatment with T3+Dex (TD) plus insulin or IGF-1. Data are mean±SEM, *n*=6 (f), *n*=8 (g), *n*=9 (h). The *n* signifies the number of wells (cell populations) measured. Statistical significance was calculated using a one-way ANOVA with Dunnett's correction, # P<0.05 for eGFP fluorescence, * P<0.05 for TMRM fluorescence.

Supplementary figure 2

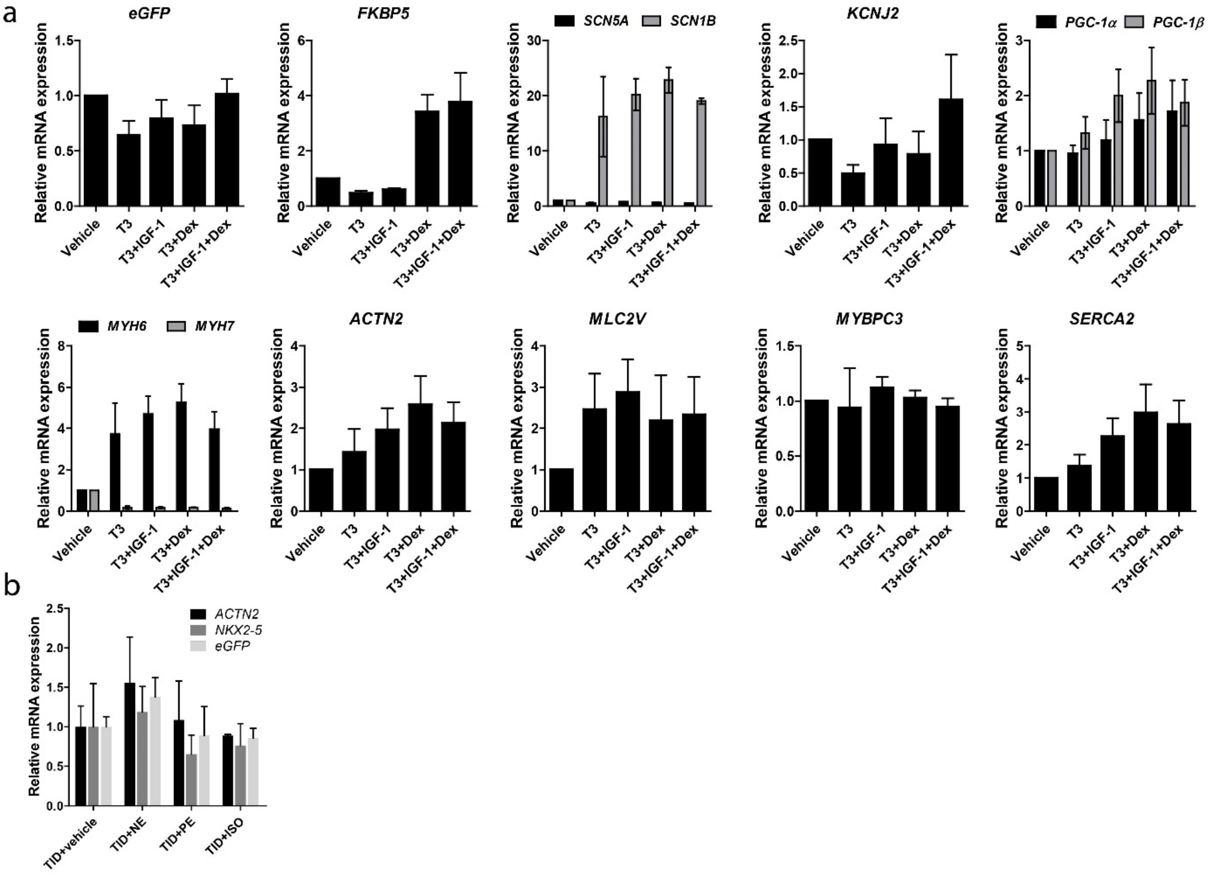

Figure S2. Gene expression profiles in hESC-derived cardiomyocytes maintained in different conditions. Related to Figure 1. (**a**) hESC-derived *NKX2-5*$^{eGFP+}$ cardiomyocytes were isolated by FACS after 7 days of treatment with the conditions shown. Gene expression is normalized to the housekeeping gene *RPLP0* and shown relative to the vehicle-only treated control. (**b**) Gene expression in cardiomyocytes treated for 5 days with T3+IGF-1+Dex (TID) plus vehicle, 1 µM norepinephrine (NE), 10 µM phenylephrine (PE) or 1 µM isoproterenol (ISO). Gene expression is normalized to the housekeeping gene *RPLP0* and shown relative to the TID+vehicle treated control. Data are mean±SEM from three independent experiments in **a**, and mean±SD for 3 replicate wells in **b**.

Supplementary figure 3

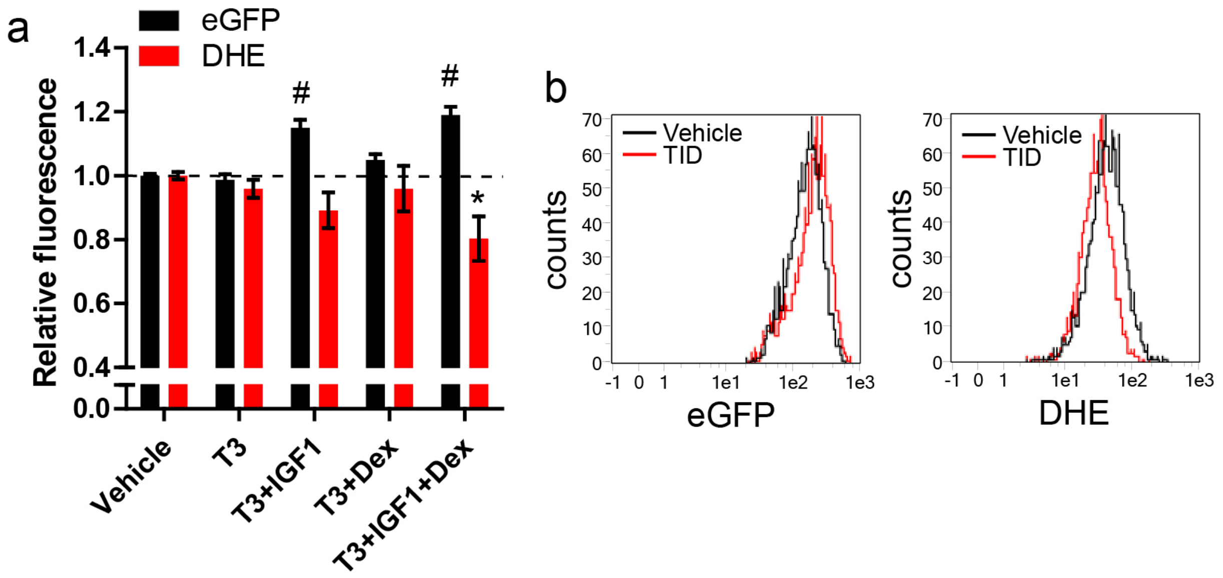

Figure S3. Modulation of reactive oxygen species levels in hESC-derived cardiomyocytes. Related to Figure 2. (**a**) Dihydroethidium (DHE) fluorescence measurements reporting superoxide radical levels in eGFP^{+} cardiomyocytes shown relative to a vehicle-only control after 5 days of maintenance with the factors shown. (**b**) Histograms of eGFP and DHE fluorescence intensity in eGFP^{+} cells maintained with vehicle-only or T3+IGF-1+Dex (TID). Note that eGFP is slightly increased while DHE is decreased by TID. Data are mean±SEM from three independent experiments. Statistical significance was calculated using a one-way ANOVA with Dunnett's correction, # P<0.05 for eGFP fluorescence, * P<0.05 for DHE fluorescence.

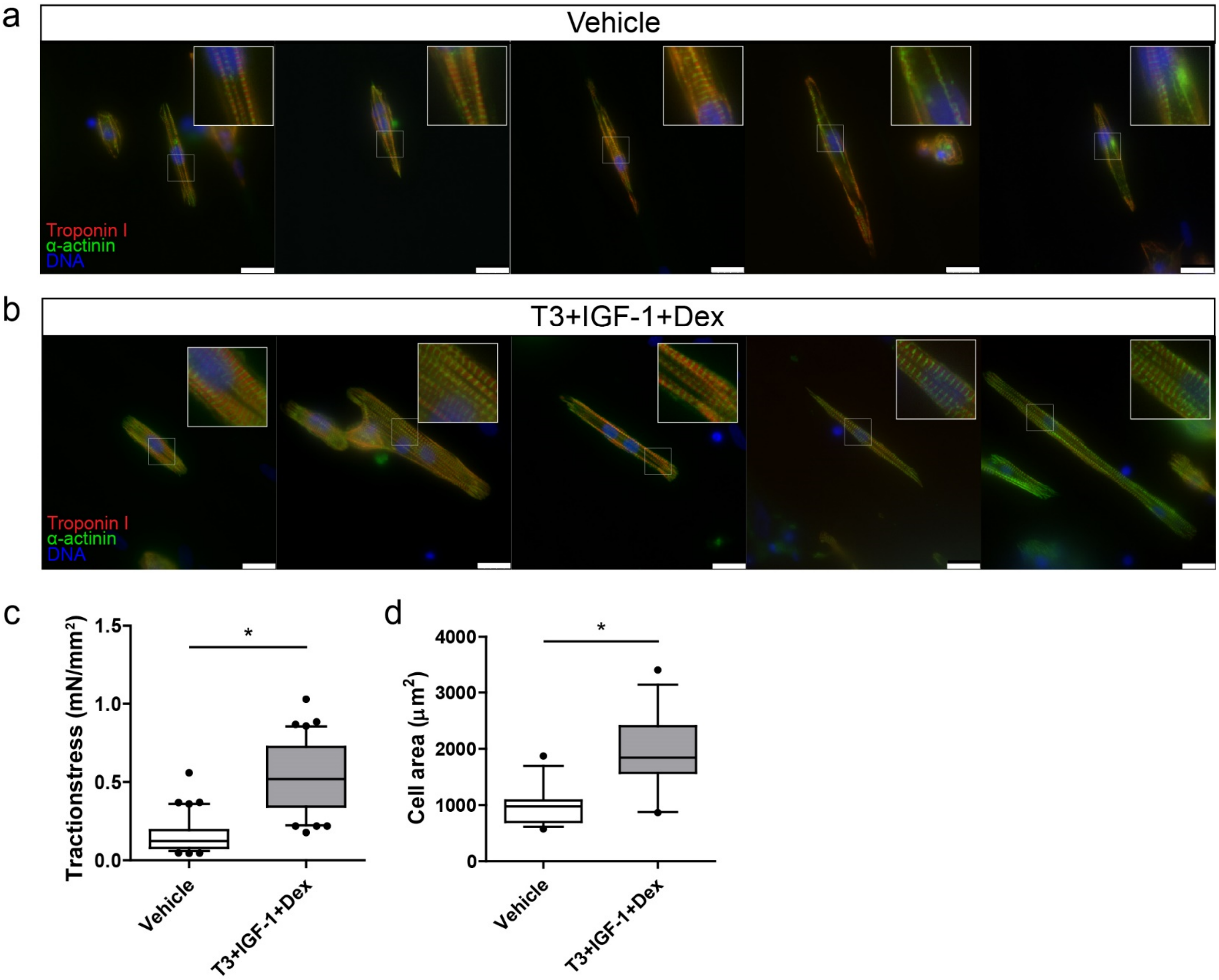

Figure S4. Structural imaging of hESC-derived cardiomyocytes and traction force analysis in an additional cell line. Related to Figure 4. (**a**) Immunostaining of Troponin I and α-actinin in cardiomyocytes maintained in (**a**) vehicle-only and (**b**) TID-medium, aligned on micropatterned PDMS gels. (**c**) Traction stress and (**d**) cell area of M1 hESC-derived cardiomyocytes maintained in vehicle or TID medium (*n*=15 individual cell measurements for each condition). Box and whisker plots show the median, interquartile range and 10-90 percentile range. Statistical significance was calculated with an unpaired t-test, * $P<0.05$.

Supplementary figure 5

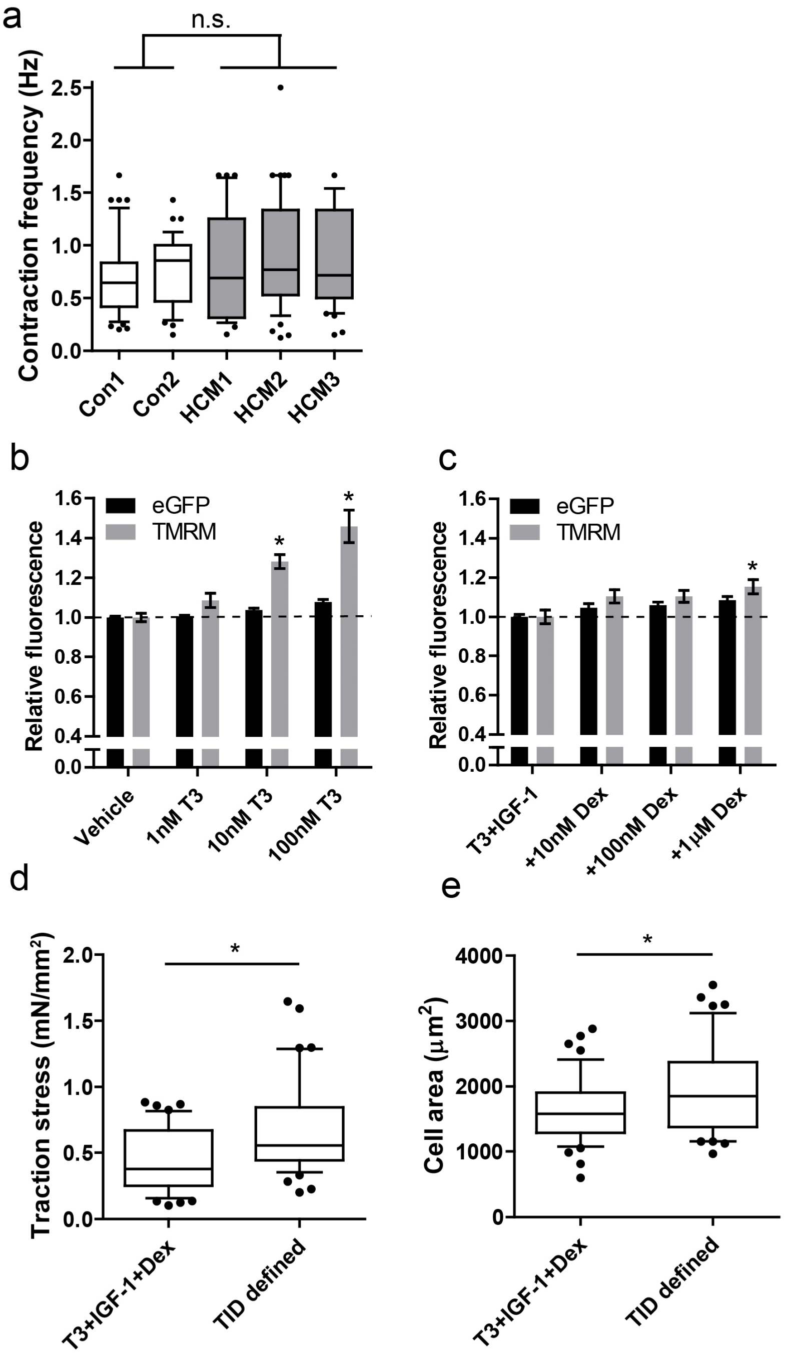

Figure S5. HCM cardiomyocyte frequency analysis and testing TID in a defined, albumin-free, basal medium. Related to Figure 5. (a) Contraction frequency of single spontaneously contracting iPSC-derived cardiomyocytes from two control (Con1 [*n*=47] and Con2 [*n*=36]) and three *MYBPC3* mutation lines (HCM1 [*n*=44], HCM2 [*n*=54] and HCM3 [*n*=43]). Box and whisker plots show the median, interquartile range and 10-90 percentile range. (**b**) eGFP and TMRM fluorescence values in eGFP^{+} cardiomyocytes relative to a vehicle-only control after 5 days of incubation with the concentrations of T3 shown (*n*=6). (**b**) eGFP and TMRM fluorescence values in eGFP^{+} cardiomyocytes maintained in T3

(100 nM) +IGF-1 supplemented with a range of dexamethasone concentrations, measured relative to the T3+IGF-1+vehicle control (T3+IGF-1). (**d**) Traction stress and (**e**) cell size of single spontaneously contracting cardiomyocytes maintained in T3+IGF-1+Dex (*n*=45) and T3+IGF-1+Dex in defined medium (*n*=43). The *n* signifies independent experimental replicates in **b** and **c** and the number of individual cells in **a**, **d** and **e** acquired over three independent experiments. Statistical significance was calculated with a one-way ANOVA with Tukey's multiple comparison test in **a**, a one-way ANOVA with Dunnett's correction in **b** and **c**, and an unpaired t-test in **d** and **e**. n.s. = not significant, * P<0.05.

Table S1. Clinical Data of HCM patients with 2373insG mutation in *MYBPC3.* Related to Figure 5.

Patient	Age	Sex	Interventricular Septum in Diastole, mm	LVEDD, mm	LVESD, mm	% FS	LVEF%
HCM1	44	m	13	54	32.9	39	56
HCM2	14	m	35	41	24	41	58
HCM3	42	m	24	55	42	24	48
Healthy Range			<13	36-56	20-40	25-43	55-70

LVEDD = left ventricular end diastolic diameter; LVESD = left ventricular end systolic diameter; % FS = fraction shortening (LVEDD-LVESD)/LVEDD*100%; LVEF% = left ventricular ejection

Supplemental Experimental Procedures (refers to Experimental Procedures)

hPSC culture and differentiation

H3 *NKX2-5*$^{eGFP/w}$ hESCs or M1 NKX2-5eGFP/w hESCs as previously generated (Elliott et al., 2011), were maintained on mouse embryonic fibroblasts and passaged using TrypLE select (Life Technologies). The generation of transgene-free hiPSCs from skin fibroblasts of one healthy male donor (LUMC0004iCtrl [Con1]) and three patients each with a c.2373dupG mutation in *MYBPC3* (LUMC0033iMyBPC [HCM1], LUMC0034iMyBPC [HCM2] and LUMC0035iMyBPC [HCM3]) was previously reported (Dambrot et al., 2014). A second transgene-free control hiPSC line (LUMC0047iCtrl [Con2]) generated from another healthy male donor was included in this study. hiPSCs were maintained on Matrigel (growth factor reduced; Corning 354230) in mTeSR1 medium (Stem Cell Technologies) and passaged with 1 mg/ml Dispase (Life Technologies).

Cardiac differentiation was induced from monolayer cultures on Matrigel in serum-free medium (BSA, polyvinyl alcohol, essential lipids [BPEL]) as previously described (Ng et al., 2008). The formulation was as follows: 44% IMDM, 44% Ham's F12, 5% protein-free hybridoma medium (PFHM-II), 0.25% BovoStar BSA (Bovogen, Australia), 0.125% polyvinyl alcohol, 1x chemically defined lipids, 400 µM α-thioglycerol (Sigma-Aldrich), 50 µg/ml L-ascorbic acid 2-phosphate (AA-2P), 2mM Glutamax, 0.1x Insulin-transferrin-selenium-ethanolamine (ITS-X) and 0.5% Pen/Strep. All BPEL components were obtained from Life Technologies unless otherwise stated. The following factors were present for the first 3 days 20 ng/ml BMP4 (R&D), 20 ng/ml Activin A (Miltenyi Biotech) and 1.5 µM CHIR 99021 (Axon Medchem). 5 µM XAV 939 (Tocris Bioscience) was present on days 3-6. *NKX2-5*$^{eGFP/w}$ hiPSCs (RD, CLM unpublished) were maintained in Essential 8 medium (Life Technologies) and differentiated as previously described (van den Berg 2015).

Contracting cultures were dissociated on day 13 and replated on Matrigel-coated 24-well plates. The following experimental factors were added on day 16, refreshed on day 20 and measured on day 21: 100 ng/ml Long R3 IGF-1 (in the main text: IGF-1), 1 µM SAG (Millipore), 1 µM dexamethasone, 100 nM triiodothyronine hormone, 10 µM phenylephrine, 1 µM isoproterenol, 1-1000 nM norepinephrine. The above factors were obtained from Sigma Aldrich unless otherwise stated.

The defined cardiomyocyte medium used in the *MYBPC3* shRNA experiment was composed of the following: DMEM (Sigma D5030), 15 mM glucose, 0.5 mM sodium pyruvate, 0.19 mM sodium hydroxybutyrate, 0.5 mM L-carnitine, 1 mM creatine, 5 mM taurine, phenol red (0.011 g/l), 1x Trace elements (A, B and C; Corning), 1x chemically defined lipids (Life Technologies), 2 mM Glutamax, 400 µM α-thioglycerol, 0.1x ITS-X, 50 µg/ml AA-2P, 0.5% Pen-Strep, 3.5 g/l sodium bicarbonate, 100 nM T3, 100 ng/ml Long R3 IGF-1 and 1 µM dexamethasone.

Lentiviral transduction

shRNAs against *MYBPC3* (NM_000256) were obtained from Open Biosystems in the pLKO Puro vector (#1 - TRCN0000082906 (AGCCAGAATATGGTTGGCTTT) and #2 - TRCN0000082903 (CCTCCCTACTGTTGGATGTAT). A scrambled shRNA was used as control (Sarbassov et al., 2005) (Addgene plasmid: 1864). Cardiomyocytes were transduced with lentiviruses on day 15 of differentiation and subsequently selected with puromycin for 4 days. Cultures were maintained for an additional 7 days before single cell dissociation onto the gelatin-patterned acrylamide gels for measurement 3 days later. Protein knockdown was assessed by Western blot.

Patterned Polyacrylamide gel fabrication

Patterned polyacrylamide gels were prepared as previously described (Rape et al., 2011). Briefly, a 1% gelatin solution was activated with 3.5 mg/mL Sodium Periodate (both Sigma-Aldrich). A polydimethylsiloxane (PDMS) stamp was casted from a SU8 master produced by standard soft lithography techniques and incubated with the activated 1% gelatin solution for 45 mins. The excess of gelatin was removed with a nitrogen gun and the stamp was used to µcontact-print a pattern of 20 µm thick with 20 µm spacing gelatin lines onto 10mm (electrophysiology) or 15 mm (contraction assay) coverslips. The polyacrylamide solution was prepared with a final concentration of 0.1% bis-acrylamide (Bio-Rad), 5% acrylamide (Bio-Rad) and 10 mM HEPES pH 8.5 in distilled water, followed by centrifugation for 1 min at 10.000 RPM for degassing. 0.006% (m/v) of ammonium persulfate (Sigma-Aldrich) and a 1:1000 dilution of 0.2-µm fluorescent beads (Ex/Em: 660/680nm - Molecular Probes) were added to the solution (for the contraction assay) and briefly vortexed. The gel polymerization was initiated with TEMED (Bio-Rad) and 4.08 µl/9.2 µl of the final solution was added to a 15 mm/25 mm coverslip treated with plus Bind-Silane solution (GE Healthcare). The µcontact-printed coverslip was applied on top of the drop with the gelatin lines facing the gel. After 20mins of polymerization the 10 mm/15 mm coverslip was removed. Each 25mm coverslip was mounted onto a well of a glassbottom 6-well plate (Mattek), replacing the initial glass. The 15mm coverslips were used directly. Plates were UV-sterilized and re-hydrated with culture medium for 30 mins before use. The polymerized gel has a Young's modulus of 5.8 kPa (Frey et al., 2007).

Flow cytometry measurements for plasma membrane potential and reactive oxygen species

For relative plasma membrane potential measurement, differentiated cultures were dissociated on day 21 (after treatment as above from day 16) using 5x TrypLE Select and resuspended in 2.5 nM TMRM (Life Technologies) in warm assay medium. Cells were incubated for 8 minutes at 37°C before being measured by flow cytometry. eGFP and TMRM fluorescence intensities were recorded. For superoxide detection, dissociated cells were labelled with 5 µM DHE for 30 mins at 37°C and then measured by flow cytometry. Samples were measured with a MACSQuant VYB (Miltenyi Biotech)

equipped with a blue (488nm) and yellow (561nm) laser. eGFP and DHE fluorescence intensities were recorded. Appropriate compensation to correct from cross-bleed was performed for each.

Respiration and acidification rates measured with the Seahorse XF-24 Analyzer

Respiration and acidification rates were measured on adherent cells using a Seahorse XF-24 or an XF-96 Analyzer (Seahorse Bioscience). The assay plates were coated with Matrigel (1:100). Cells were seeded in BPEL medium at a density of $6.5x10^4$ (XF-24) or $2.5x10^4$ (XF-96) cells/well 7 days before measurement. The assay was performed in bicarbonate-free DMEM (D5030; Sigma Aldrich), containing 0.25% fatty acid-free BovoStar (Bovogen, Australia), 1 mM sodium lactate, 0.15 mM sodium hydroxybutyrate, 2 mM GlutaMAX (Life Technologies) and 0.5 mM L-carnitine. The standard glucose concentration was 15 mM glucose and was supplemented with 0.5 mM sodium pyruvate. Cells were washed twice and pre-incubated in the assay medium for 1 h before measurement. For the standard profiling oligomycin was used at 0.5 µg/ml, FCCP titrated in 2 injections to 3 µM and rotenone and antimycin A were added at 1 µM and 2 µM respectively. A standard protein assay was used to normalize values to whole cell protein. Acid titration experiments were performed to calculate the buffering capacity of the Seahorse assay media and gave a value of 0.1 pmoles H+/mpH in the 7 µl measuring volume. ECAR values were converted to anaerobic glycolyis/lactate production rates as previously described (Mookerjee et al., 2015), using a max H+/O2 value of 1.0 and converted 1:1 to an ATP production rate. The oligomycin-sensitive oxygen consumption was converted to an ATP production rate using a P/O ratio of 2.3 (Brand, 2005). To assess ATP demand for excitation and contractility nifedipine (10 µM) and blebbistatin (5 µg/ml) were co-injected and the respiration rate immediately recorded, followed by measurements after oligomycin and then rotenone and antimycin A injection. A vehicle-only injection was performed in parallel and the effect subtracted. Experiments analysing the effect of etomoxir and UK5099 were performed on the XF-96 format using an assay medium with the following modifications: the glucose concentration was 5.5 mM, sodium pyruvate 0.15 mM and palmitic acid was included at 100 µM conjugated to fatty acid-free BovoStar BSA in a 2.8:1 molar ratio. To prepare this a 4 mM palmitic acid solution was made in assay medium by heating at 65°C for 30 mins, then combined 1:1 with a 10% Bovostar BSA solution and heated at 37°C for 30 mins with mixing. 10 mM HEPES was also included in this assay medium to provide extra buffering (glycolytic rates were not calculated). Cells were washed twice and pre-incubated in this assay medium for 4 h before measurement. To assess the response to substrate uptake inhibitors, 40 µM etomoxir or 5 µM UK5099 were injected and respiration rates recorded after 45 mins, followed by injections of oligomycin, FCCP (3 µM) and then rotenone and antimycin A. A vehicle-only injection was performed in parallel and the effect subtracted.

Electrophysiological characterization

Action potential (AP) recordings were performed on single cardiomyocytes, 14-16 days after cell dissociation with the amphotericin perforated patch-clamp technique using an Axopatch 200B amplifier (Molecular Devices Corporation, Sunnyvale, CA, USA). Signals were filtered and digitized at 5 and 40 kHz, respectively. Data acquisition and analysis were accomplished using pClamp10.1 (Axon Instruments) and custom software. Potentials were corrected for the liquid junction potential. Cells were continuously perfused in a perfusion chamber at 37 ^{0}C (Cell MicroControls Norfolk VA, U.S.A.) using Tyrode's solution containing (mM): NaCl 140, KCl 5.4, CaCl2 1.8, MgCl2 1.0, glucose 5.5, HEPES 5; pH 7.4 (NaOH). Pipettes (borosilicate glass; resistance ~2.5 MΩ) were filled with solution containing (mM): K-gluconate 125, KCl 20, NaCl 5, amphotericin-B 0.22, HEPES 10; pH 7.2 (KOH).

APs were recorded at spontaneous frequencies and characterized by duration at 50 and 90% repolarization (APD50, and APD90, respectively), maximal diastolic potential (MDP), AP amplitude, maximal upstroke velocity and frequency. AP parameter values obtained from 8-9 consecutive APs were averaged and data were collected from at least 2 independent differentiations per condition.

Traction force measurements

The traction force measurements were performed as previously described (Hazeltine et al., 2012). Cells were seeded on gelatine patterned acrylamide gels 4 days before and measured in their normal culture medium in an environment maintained at 37°C with 5% CO_2. Briefly, using a Leica AF-6000LX microscope, an image-series of aligned single spontaneously contracting cardiomyocytes was taken at 40x magnification at 20 frames per second, recording brightfield and fluorescent beads. Single frames from maximal relaxation and contraction of the brightfield and fluorescent beads image-series were analyzed by the LIBTRC software package (kindly provided by Dr. Micah Dembo), creating a mask of the cell outline from the brightfield image and a vector map from the difference between the relaxed and contracted fluorescent beads images. The vector map and the cell mask were used to calculate the maximum total force that the cell applies on the substrate at its peak of contraction. The traction stress generated by the cardiomyocyte during contraction was calculated by dividing the total force by the cell surface area. Measurements were blinded in acquisition and analysis.

Quantitative real-time PCR

RNA was isolated using a Minelute RNA extraction kit (Qiagen) and cDNA synthesized using an iScript cDNA synthesis kit (BioRad). Real-time PCR was performed on a BioRad CFX384 machine using IQ SYBR Green (BioRad). Gene expression values were normalized to the mean expression of the housekeeping genes human ribosomal protein (*RPLP0*), glucuronidase (*GUSB*), and *RNF7*. Primer sequences were as follows:

Gene	Forward primer	Reverse primer
RPLP0	CACCATTGAAATCCTGAGTGATGT	TGACCAGCCCAAAGGAGAAG
GUSB	CCACCTAGAATCTGCTGGCTAC	GTGCCCGTAGTCGTGATACCAA
RNF7	ACGCACCGAATAGTTACGG	CCAGGTCCACGGGCAGA
eGFP	GTGAGCAAGGGCGAGGAG	CCGTAGGTCAGGGTGGTC
FKBP5	TTCTCCTTGCTGCCTTTCTG	ACCCTTGGCTGACTCAAACTC
SCN5A	GAGCTCTGTCACGATTTGAGG	GAAGATGAGGCAGACGAGGA
SCN1B	GTGGTTGTAGGTGACATTGGTG	GAAGGGCACTGAGGAGTTTGT
KCNJ2	ACCGCTACAGCATCGTCTCT	TCCACACACGTGGTGAAGAT
PGC-1α	AACACTTACAAGCCAAACCA	GGGTTCAATAGTCTTGTTCTC
PGC-1β	TCCTCAACTATCTCGCTGAC	CTCACTGTCAATCTGGAAGAG
MYH6	CTTCTCCACCTTAGCCCTGG	GCTGGCCCTTCAACTACAGA
MYH7	CGACCTTCTTCTCTTGCTC	GAGGACAAGGTCAACACCCT
ACNT2	CTGCTGCTTTGGTGTCAGAG	TTCCTATGGGGTCATCCTTG
MYL2/MLC2V	TACGTTCGGGAAATGCTGAC	TTCTCCGTGGGTGATGATG
MYBPC3	GGCATGCTAAAGAGGCTCAA	TCTTGTGGCCTTTGCTCAC
SERCA2	ACCCACATTCGAGTTGGAAG	CCAACGAAGGTCAGATTGGT

Western blotting

The samples were lysed with ice cold ELB (50mM HEPES pH 7.0; 250mM NaCl; 5mM EDTA; 0.1% NP-40) with 1:100 Protease Inhibitor Cocktail (Sigma-Aldrich) for 30 mins on ice. The samples were centrifuged at 7,000g for 10 mins and the supernatant was quantified for protein content using the Bio-Rad Protein Assay (based on the Bradford dye-binding method). 100µg of the hiPSC-CM lysates were run in a 6% polyacrylamide gel and 30µg of the hESC-CM with the shRNA lysates were run in a 10% polyacrylamide gel together with the protein ladder (Precision Plus Protein dual colour – Bio-Rad). The protein was transferred to an Amersham Hybond membrane (GE Heathcare- Life Sciences) overnight. The membrane was blocked with blocking buffer (2% milk in PBS) and each protein was detected with the specific antibody: Rabbit IgG anti- cMyBP-C (1:2000) kindly provided by Dr. Sakthivel Sadayappan from the Department of Cell and Molecular Physiology, Health Sciences Division, Loyola University Chicago, followed by Horse anti-Rabbit IgG – HRP (1:2000) (Cell Signaling). Mouse IgG anti-Actin (1:1000) (MAB1501 – Millipore) and Mouse IgG anti-α-actinin (A7811-Sigma-Aldrich), followed by Goat anti-mouse IgG-HRP (1:2000) (Cell Signaling).

Supplemental references

Frey, M.T., Engler, A., Discher, D.E., Lee, J., and Wang, Y.-L. (2007). Microscopic methods for measuring the elasticity of gel substrates for cell culture: microspheres, microindenters, and atomic force microscopy. Methods Cell Biol. *83*, 47–65.

Rape, A.D., Guo, W.-H., and Wang, Y.-L. (2011). The regulation of traction force in relation to cell shape and focal adhesions. Biomaterials *32*, 2043–2051.
