# Supplementary figures and images for: Contractile Defect Caused by Mutation in MYBPC3 Revealed under Conditions Optimized for Human PSC-Cardiomyocyte Function
Source: Cell Rep. 2015 Oct 17;13(4):733–45. doi: 10.1016/j.celrep.2015.09.025 (PMC4644234; doi:10.1016/j.celrep.2015.09.025)

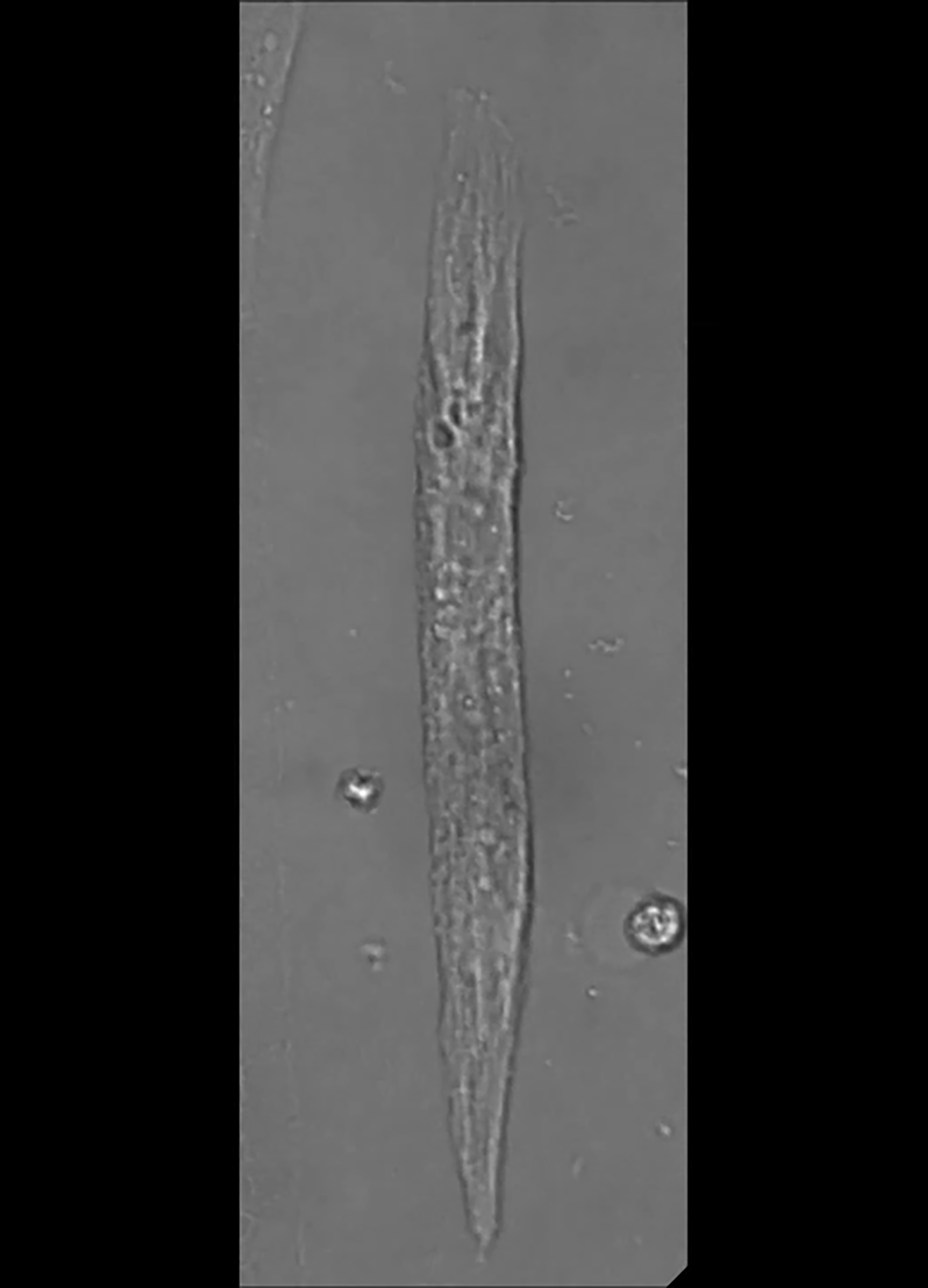

Supplement: Movie S1. Typical Spontaneously Contracting hESC Cardiomyocytes on Micropatterned Polyacrylamide in Control Medium or TID-Containing Medium, Related to Figure 4 [file mmc2.jpg]

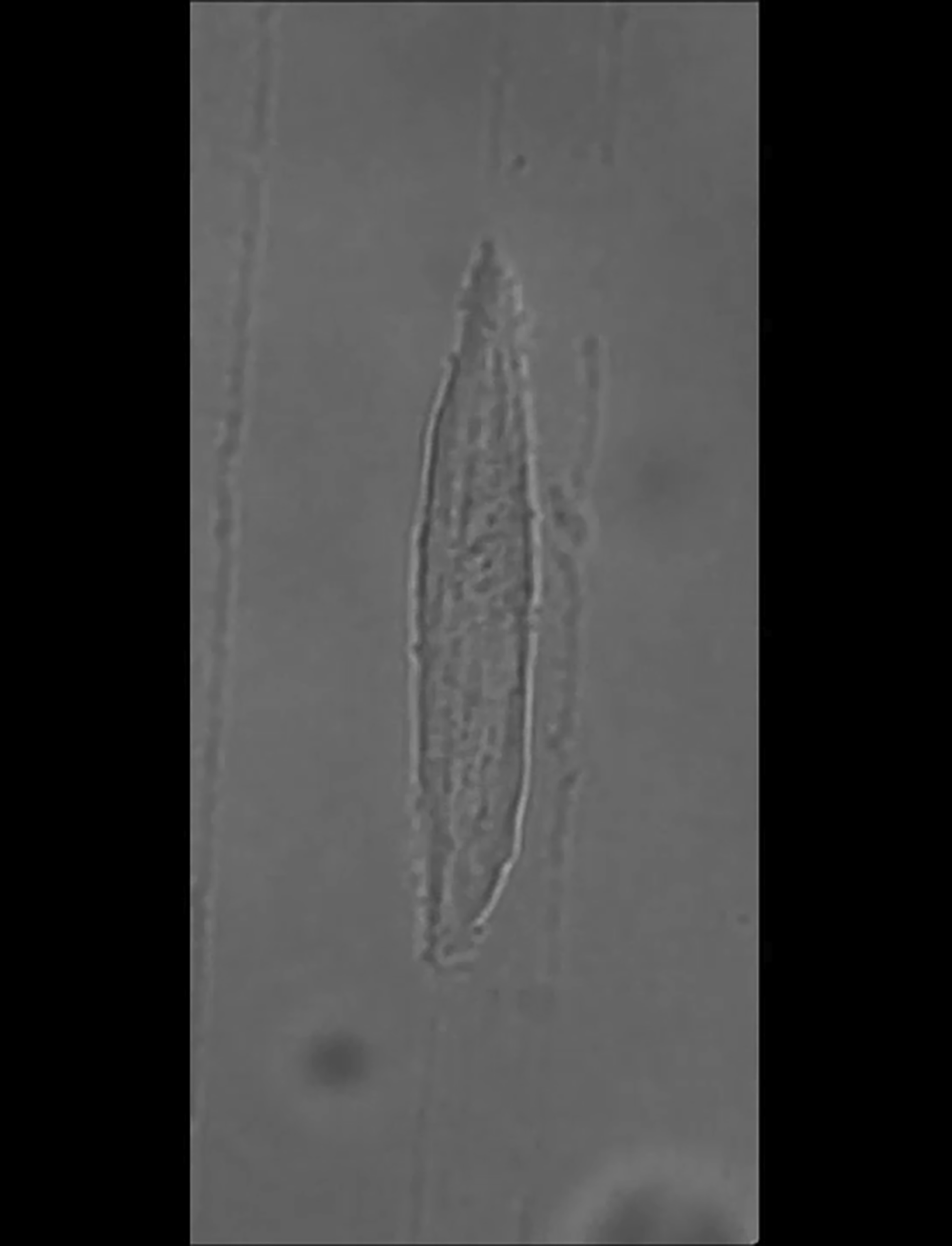

Supplement: Movie S2. Typical Spontaneously Contracting hiPSC Cardiomyocytes, Control, or HCM on Micropatterned Polyacrylamide in TID-Containing Medium, Related to Figure 5 [file mmc3.jpg]
