# Supplementary material for: Contractile Defect Caused by Mutation in MYBPC3 Revealed under Conditions Optimized for Human PSC-Cardiomyocyte Function
Source: Cell Rep. 2015 Oct 17;13(4):733–45. doi: 10.1016/j.celrep.2015.09.025 (PMC4644234; doi:10.1016/j.celrep.2015.09.025)
Supplement: Document S2. Article plus Supplemental Information [file mmc4.pdf]

Cell Reports

Article

Contractile Defect Caused by Mutation in *MYBPC3* Revealed under Conditions Optimized for Human PSC-Cardiomyocyte Function

Graphical Abstract

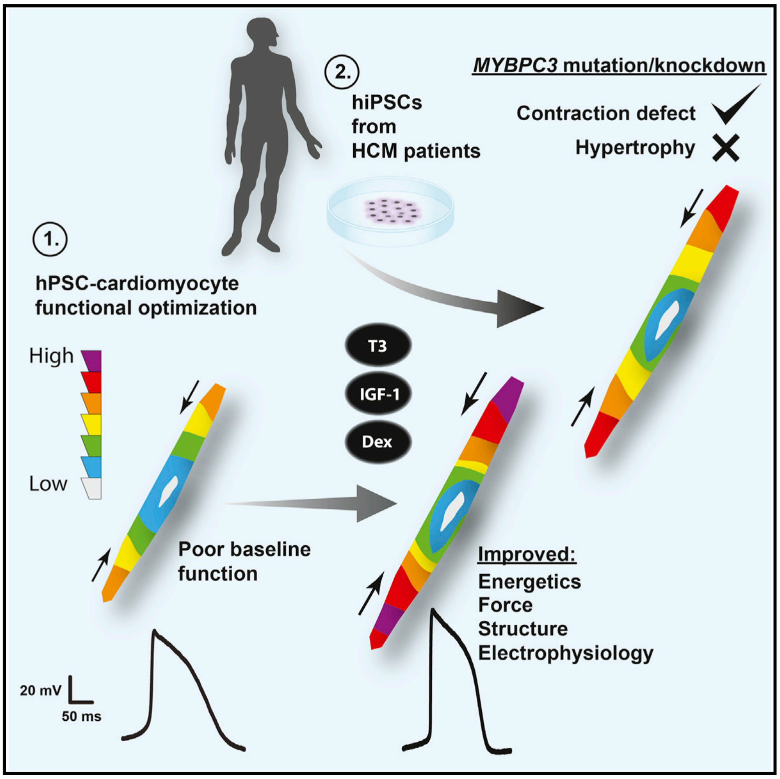

Highlights

- $T3^{+}IGF-1^{+}$ dexamethasone improves the electrophysiology of hPSC cardiomyocytes
- These factors synergistically enhance bioenergetics and contractile force generation
- Cardiomyocytes with HCM-causing mutations have a contractile defect

Authors

Matthew J. Birket, Marcelo C. Ribeiro, Georgios Kosmidis, ..., Douwe E. Atsma, Robert Passier, Christine L. Mummery

Correspondence

c.l.mummery@lumc.nl

In Brief

Birket et al. identify a combination of factors that cooperatively improve the function of human pluripotent stem cell (hiPSC)-derived cardiomyocytes. Optimizing the system facilitated the identification of a contraction force defect in a model of hypertrophic cardiomyopathy (HCM), a disease affecting ~1:500 of the population.

Birket et al., 2015, Cell Reports *13*, 733–745
October 27, 2015 ©2015 The Authors
http://dx.doi.org/10.1016/j.celrep.2015.09.025

Cell Reports
Article

OPEN ACCESS
CellPress

Contractile Defect Caused by Mutation in *MYBPC3* Revealed under Conditions Optimized for Human PSC-Cardiomyocyte Function

Matthew J. Birket,[1,4] Marcelo C. Ribeiro,[1,4] Georgios Kosmidis,[1] Dorien Ward,[1] Ana Rita Leitoguinho,[1] Vera van de Pol,[1] Cheryl Dambrot,[1,2] Harsha D. Devalla,[1] Richard P. Davis,[1] Pier G. Mastroberardino,[3] Douwe E. Atsma,[2] Robert Passier,[1,5] and Christine L. Mummery[1,5,*]
[1]Department of Anatomy and Embryology, Leiden University Medical Center, 2300 RC Leiden, the Netherlands
[2]Department of Cardiology, Leiden University Medical Center, 2300 RC Leiden, the Netherlands
[3]Erasmus Medical Center, 3015 GE Rotterdam, the Netherlands
[4]Co-first author
[5]Co-senior author
*Correspondence: c.l.mummery@lumc.nl
http://dx.doi.org/10.1016/j.celrep.2015.09.025
This is an open access article under the CC BY license (http://creativecommons.org/licenses/by/4.0/).

SUMMARY

Maximizing baseline function of human pluripotent stem cell-derived cardiomyocytes (hPSC-CMs) is essential for their effective application in models of cardiac toxicity and disease. Here, we aimed to identify factors that would promote an adequate level of function to permit robust single-cell contractility measurements in a human induced pluripotent stem cell (hiPSC) model of hypertrophic cardiomyopathy (HCM). A simple screen revealed the collaborative effects of thyroid hormone, IGF-1 and the glucocorticoid analog dexamethasone on the electrophysiology, bioenergetics, and contractile force generation of hPSC-CMs. In this optimized condition, hiPSC-CMs with mutations in *MYBPC3*, a gene encoding myosin-binding protein C, which, when mutated, causes HCM, showed significantly lower contractile force generation than controls. This was recapitulated by direct knockdown of *MYBPC3* in control hPSC-CMs, supporting a mechanism of haploinsufficiency. Modeling this disease in vitro using human cells is an important step toward identifying therapeutic interventions for HCM.

INTRODUCTION

Human pluripotent stem cells (hPSCs) are becoming increasingly used in biomedical research. Their extensive growth and differentiation potential enables routine production of cell types otherwise difficult to obtain. Cardiomyocytes are an important example, since collecting biopsies from the heart is highly invasive, yet the cells they contain are those most pertinent to heart disease and drug-associated toxicity. Although many reports have now demonstrated the utility of hPSC-derived cardiomyocytes (hPSC-CMs) for modeling heart disease (Bellin et al., 2013; Drawnel et al., 2014; Wang et al., 2014; Zhang et al., 2014), the baseline functional performance of these cells is still less than that in adult heart tissue. Low maximum diastolic potential (MDP) and slow action potential upstroke velocities are frequently observed across all cardiomyocyte subtypes (Sartiani et al., 2007; Zhang et al., 2009), coupled with a low force of contraction (Hazeltine et al., 2012; Ribeiro et al., 2015).

The poor functional output of hPSC-CMs is in large part due to their developmental immaturity (Veerman et al., 2015). While efforts are ongoing to advance the developmental state beyond their fetal equivalents, another important and valid approach is to also consider ways to maximize cell function more immediately, which might not necessarily be coupled to or regulated in the same way as developmental maturation. This might be achieved, for example, by improving aspects of cell metabolism and bioenergetics. Cardiomyocytes maintained outside their native environment within the heart may be disconnected from many factors important for their basic physiology, including relevant growth factor and hormone signaling, extracellular matrix proteins, other cell populations in the heart and mechanical and electrical stimulation. As protocols for hPSC-CM generation have improved and the generation of pure populations of cardiomyocytes in serum-free media has become well established, this separation becomes even more notable. Many physiological factors that would normally be circulating in the heart are absent under most basal culture conditions, and it is unclear which should be provided in a defined formulation to promote optimal cardiomyocyte function in bioassay development.

The MDP is a fundamental determinant of excitability and in cardiomyocytes from adult "working myocardium" is around −85 mV (Magyar et al., 2000). The lack of I_{k1} current due to low expression of the *KCNJ2* gene is one reason that ventricular- and atrial-like cardiomyocytes from hPSCs are insufficiently polarized (Ma et al., 2011; Sartiani et al., 2007; Synnergren et al., 2012). However, many other factors affect the resting membrane potential. These include the activity of the energy demanding Na,K-ATPase and potassium conductance as determined by other channels, which may also not be appropriately

expressed or function optimally. The MDP is a strong predictor of contractile output in hPSC-CMs (Ribeiro et al., 2015), and so improving MDP is an important prerequisite for faithfully modeling diseases affecting excitability or contractility.

One disease predicted to affect cardiomyocyte contractility is hypertrophic cardiomyopathy (HCM). HCM is highly prevalent, affecting 1:500 of the population and can be caused by mutations in various sarcomeric protein encoding genes (Maron and Maron, 2013). Approximately 50% of cases are the result of mutations either in *MYH7*, encoding β-myosin heavy chain, or in *MYBPC3,* encoding cardiac myosin-binding protein C (cMyBP-C). As a disease with known genetic cause but ill-defined molecular pathogenesis, HCM is an excellent candidate for stem cell modeling (Eschenhagen et al., 2015). The absence of a cure or even an effective treatment strategy provides great urgency for in vitro human models to identify drugs able to restore contractile function and prevent the development of hypertrophy, cardiac remodeling, and consequential arrhythmias. Human induced pluripotent stem cells (hiPSCs) have already been generated from patients with mutations in *MYH7* and *MYBPC3*, and cardiomyocytes derived from them (Dambrot et al., 2014; Han et al., 2014; Lan et al., 2013), but their force generation, measured on physiologically relevant substrate dimensions and stiffness, has not yet been described and is currently hindered by poor baseline performance as noted above.

In this study, we began with a well-characterized *NKX2-5*$^{eGFP/w}$ human embryonic stem cell (hESC) line, in which working cardiomyocytes are specifically marked by eGFP expression (Birket et al., 2015; Elliott et al., 2011), to identify factors or combinations of factors, which promoted an increase in their resting plasma membrane potential, or that had an anabolic effect, making the assumption that such factors could have functional benefit. We then tested these factors at the level of single-cell traction force and action potential generation. Optimized conditions were then applied to an hiPSC model of cMyBP-C insufficiency, and this revealed a contractile defect in cardiomyocytes derived from *MYBPC3* mutation carriers, which was mimicked by knockdown of *MYBPC3* in hESC-derived cardiomyocytes.

RESULTS

The Identification of Factors Modulating hPSC-Derived Cardiomyocyte Plasma Membrane Potential and Size

Populations of cardiomyocytes were generated by monolayer differentiation of *NKX2-5*$^{eGFP/w}$ hESCs, and these were maintained until day 16. To screen for modulators of resting plasma membrane potential ($\Delta\Psi$p), we assessed short-term accumulation of the cationic fluorescent probe tetramethylrhodamine methyl ester (TMRM) in cell suspensions. TMRM follows Nernstian behavior across cell membranes, the early phase of TMRM accumulation into cells is dominated by the $\Delta\Psi$p, while with longer loading times the mitochondrial membrane potential and matrix volume contribute increasingly to total accumulation (Gerencser et al., 2012). Time-course measurement of TMRM accumulation was performed from control conditions (Figures 1A and 1B). Based on this, 8-min loading time was chosen for relative assessments of $\Delta\Psi$p, since this was in the linear phase of accumulation but gave signal substantially above background. Additionally, total cellular eGFP fluorescence was used as an estimate of cell size/volume, on the assumption that eGFP levels should correlate with total cell protein. There are several caveats to this reasoning, but modulators can be subsequently validated with more direct assays. In support of a general correlation, fetal calf serum exposure, known to increase the size of these cells (Birket et al., 2013), increased eGFP levels after 5 days of exposure (Figure S1).

The following factors, each with putative roles in cardiomyocyte function, were tested: insulin-like growth factor 1 (IGF-1), the hedgehog signaling agonist SAG, the synthetic glucocorticoid dexamethasone (Dex), triiodothyronine hormone (T3), the α-adrenergic agonist phenylephrine (PE), and the β-adrenergic agonist isoproterenol (ISO) (El-Armouche and Eschenhagen, 2009; Bisping et al., 2012; Clement et al., 2009; Li et al., 2014; Rog-Zielinska et al., 2015; Troncoso et al., 2014). Testing was performed in a serum-free, low-insulin medium that has become a widely used standard for maintenance of these cells (Ng et al., 2008). Contracting monolayers of differentiated cells (day 16) were maintained for 5 days in each test condition before measurement (Figure 1C). IGF-1 was the only factor to significantly increase eGFP fluorescence intensity, and TMRM accumulation increased only proportionally in this condition. IGF-1 may therefore mildly increase cell volume without affecting $\Delta\Psi$p. T3 was the only factor that significantly increased TMRM accumulation, while eGFP fluorescence remained unaffected. This indicated an increase in $\Delta\Psi$p by T3, but not in cell volume. While ISO mildly increased TMRM accumulation, neither ISO nor PE induced an increase in eGFP fluorescence; inconsistent with their established role as hypertrophic agonists in other cardiomyocyte systems but consistent with a reported variable response in some hPSC-CM lines (Földes et al., 2014; Vidal et al., 2012; Zhang et al., 2013). Dex had little effect on either eGFP or TMRM, consistent with unpublished work from our group showing a lack of effect by Dex-alone on cardiomyocyte electrophysiology in hPSC-derived cardiomyocytes (G.K., C.L.M., M. Bellin, B. van Meer, L. Tertoolen, and S. Casini, unpublished data), but inconsistent with its previously described role in cardiomyocyte maturation (Rog-Zielinska et al., 2013, 2015), suggesting that competence factors may be lacking.

These results support a role for T3 in determining $\Delta\Psi$p in hPSC-derived cardiomyocytes, but not for maturation-related growth. To explore the possibility of enhancing this condition further, we tested the effect of additional IGF-1 or Dex compared to T3-alone (assigned as the new reference control and normalized to 1) (Figure 1D). The addition of IGF-1 again mildly increased eGFP levels over T3-alone, while T3+Dex mildly, but significantly, increased TMRM accumulation. However, the combination of T3+IGF-1+Dex (TID) increased both eGFP and TMRM significantly, suggesting an enhancement in both size and possibly $\Delta\Psi$p (Figure 1E), which could be associated with further improved cardiomyocyte function. The increased eGFP fluorescence with T3+IGF-1 and TID was not associated with, and therefore explained by, a relative increase in *eGFP* mRNA expression in these cells (Figure S2). Upregulation (~4-fold) of the glucocorticoid-response gene *FKBP5* confirmed increased glucocorticoid signaling by Dex (Figure S2). The action of TID on eGFP levels and TMRM accumulation was confirmed in

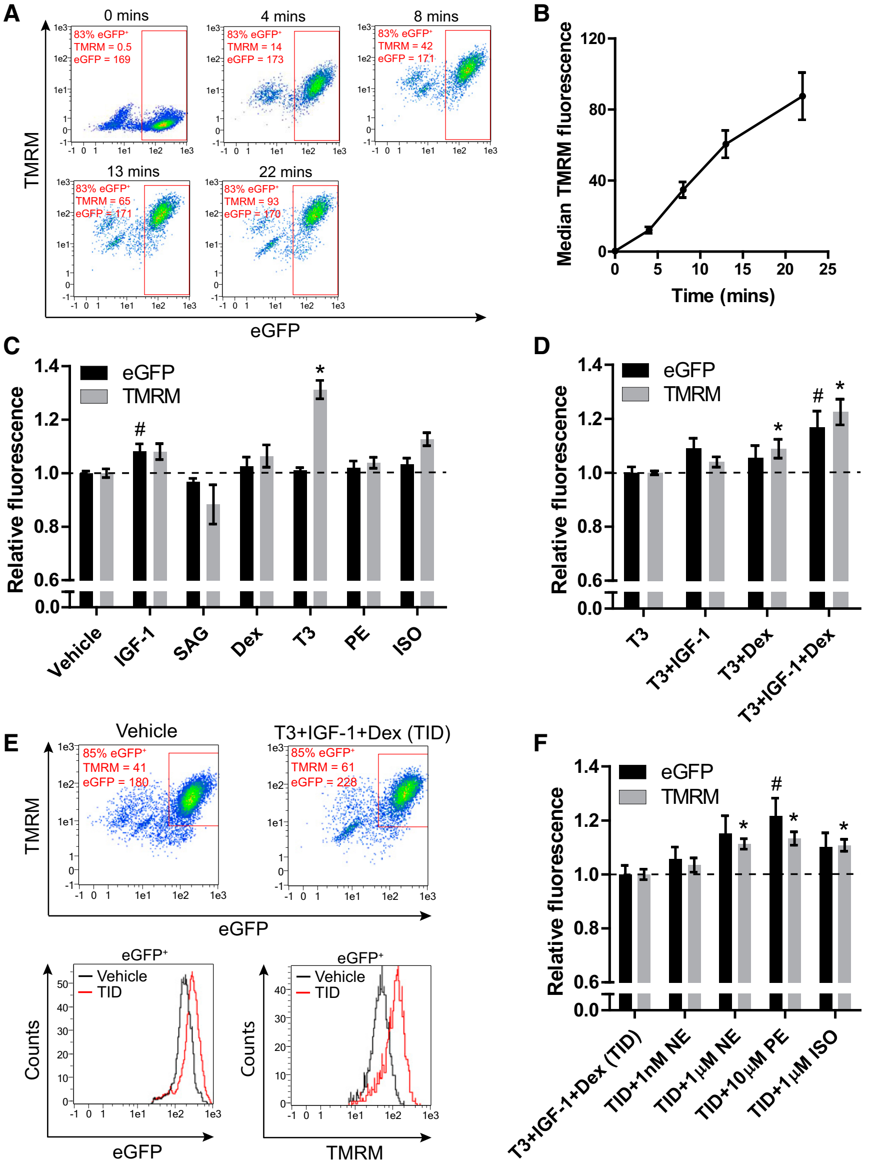

Figure 1. Using TMRM to Identify Modifiers of $\Delta\Psi p$ in hESC-Derived NKX2-5^{+} Cardiomyocytes

(A) Time-course measurement of TMRM accumulation after cardiac differentiation of *NKX2-5$^{eGFP/w}$* hESCs. fluorescence-activated cell sorting (FACS) plots show eGFP and TMRM fluorescence after 0, 4, 8, 13, and 22 min of loading with TMRM. A minor cross-bleed correction has been applied to all.

(B) Median TMRM fluorescence intensity in eGFP^{+} cells plotted against loading time.

(C) eGFP and TMRM fluorescence values in eGFP^{+} cardiomyocytes relative to a vehicle-only control after 5 days of incubation with the factors shown (n = 6–34).

(D) eGFP and TMRM fluorescence values in eGFP^{+} cardiomyocytes relative to a T3-treated control after 5 days of incubation with the factors shown (n = 10–22).

(E) Upper panel: example FACS plots showing measurement of a vehicle-only control alongside a T3+IGF-1+Dex-treated sample. Lower panel: histograms of eGFP and TMRM intensity in the eGFP^{+} populations as marked on the dot plots above.

(F) eGFP and TMRM fluorescence values in eGFP^{+} cardiomyocytes relative to a T3+IGF-1+Dex-treated sample after 5 days of incubation with the factors shown (n = 11).

Data are mean ± SEM. The n signifies biological replicates. Statistical significance compared to the vehicle-only control was calculated using a one-way ANOVA with Dunnett's correction, #p < 0.05 for eGFP fluorescence; *p < 0.05 for TMRM fluorescence. See also Figures S1 and S2.

cardiomyocytes from an independent *NKX2-5$^{eGFP/w}$* knockin reporter M1 hESC line as well as an *NKX2-5$^{eGFP/w}$* knockin reporter hiPSC line (Figure S1).

Given that insulin was present in the basal medium at 1 μg/ml for all these assays and at these concentrations can cross-react with the IGF-1 receptor, we separately tested the impact of insulin and IGF-1 in this T3+Dex condition. Addition of 1 μg/ml insulin-alone to the medium containing T3+Dex significantly increased eGFP fluorescence, but addition of 100 ng/ml IGF-1 increased eGFP fluorescence further and also increased TMRM accumulation (Figure S1). This suggests that activation of the IGF receptor is the primary mediator of this response.

Importantly, in the presence of TID, cardiomyocytes responded appropriately to stimulation by the adrenergic agonists norepinephrine (NE), PE, or ISO, with an increase in eGFP fluorescence, suggesting that a baseline level of bioenergetic or anabolic activity may be important for this hypertrophic response (Figure 1F). Relative *eGFP* mRNA expression was not significantly affected by these factors (Figure S2). The action of adrenergic agonists did not, however, increase TMRM accumulation beyond the increase in eGFP intensity, suggesting increased TMRM was probably due to increased cell volume and not due to a further improvement in $\Delta\Psi p$ by these factors.

Bioenergetic Response in Cardiomyocytes by the Concerted Action of T3, IGF-1, and Dexamethasone

The response to IGF-1 and Dex in terms of cardiomyocyte growth and $\Delta\Psi p$ suggests a possible synergistic role in metabolic stimulation. To test this, bioenergetic profiling of contracting cardiomyocyte monolayers was performed using the Seahorse XF Analyzer on cells treated during the same experimental time course. Contraction rates, measured prior to analysis, were not significantly different between groups (Figure 2A). Under standard conditions (15 mM glucose, 0.5 mM sodium pyruvate) normalized to total cell protein, T3 mildly increased the oligomycin-sensitive respiration rate as previously reported (Yang et al., 2014), indicating increased mitochondrial ATP

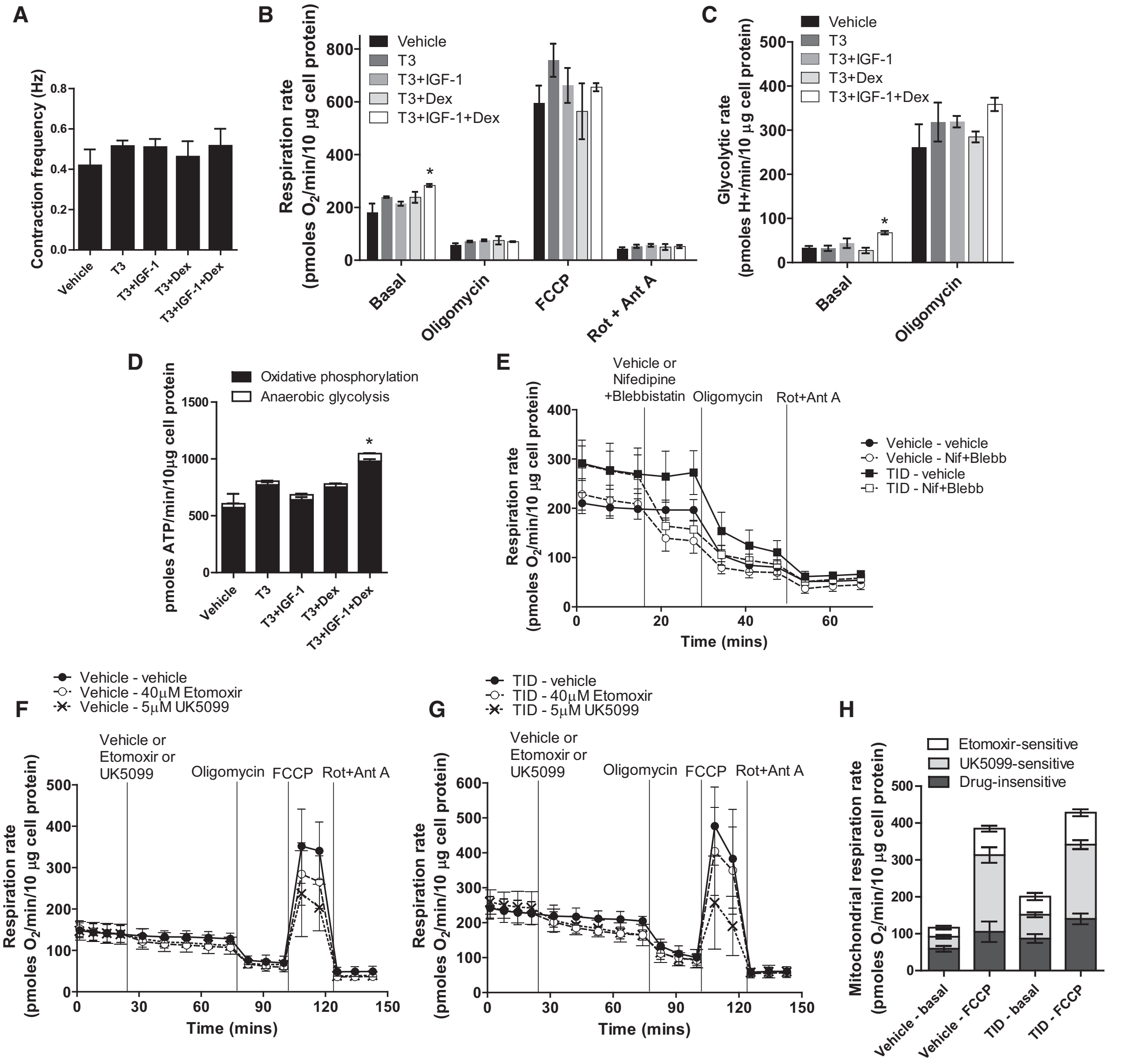

Figure 2. Bioenergetic Profiling of hESC-Derived Cardiomyocytes

(A) Contraction frequency in cardiomyocyte monolayers, treated for 5 days with the factors shown, prior to Seahorse measurement.

(B) Respiration rates in cardiomyocyte monolayers treated with the factors shown, normalized to cell protein. Basal, endogenous rate; oligomycin, ATP synthase-inhibited rate; FCCP, maximum uncoupled rate; Rot + Ant A, non-mitochondrial respiratory rate.

(C) Glycolytic rate measured in parallel with respiration, normalized to cell protein.

(D) Theoretical basal ATP production rates from oxidative phosphorylation and anaerobic glycolysis calculated from measurements in (B) and (C).

(E) Real-time respiration measurements of vehicle-only and TID-treated cells and response to injection of vehicle-only or nifedipine + blebbistatin, then oligomycin, and finally rotenone and antimycin A.

(F and G) Real-time respiration measurements of (F) vehicle-only or (G) TID-treated cells and response to injection of vehicle-only or 40 μM etomoxir or 5 μM UK5099, then oligomycin, FCCP, and finally rotenone and antimycin A.

(H) Sensitivity of basal and FCCP-stimulated mitochondrial respiration to etomoxir and UK5099 in vehicle-only or TID-treated cells.

Bar data are mean ± SEM from three independent experiments, each comprising four to five measurement wells per condition. Real-time respiration plots show data of a typical experiment mean ± SD of individual wells. Statistical significance compared to the vehicle-only control was calculated using a one-way ANOVA with Dunnett's correction for (B)–(D). *$p < 0.05$. See also Figure S3.

turnover (Figures 2B and 2D). Neither IGF-1 nor Dex stimulated this further, but the combination of TID did have a further stimulatory effect (TID: 213 ± 4 versus vehicle: 124 ± 6 pmol O_2/min/10 μg cell protein; $p < 0.05$). The anaerobic glycolytic rate (calculated as previously described by Mookerjee et al., 2015) was also significantly increased by TID (TID: 68 ± 4 versus vehicle: 34 ± 4 pmol H^+/min/10 μg cell protein; $p < 0.05$) (Figure 2C). A conversion of these values to ATP production rates (see Experimental

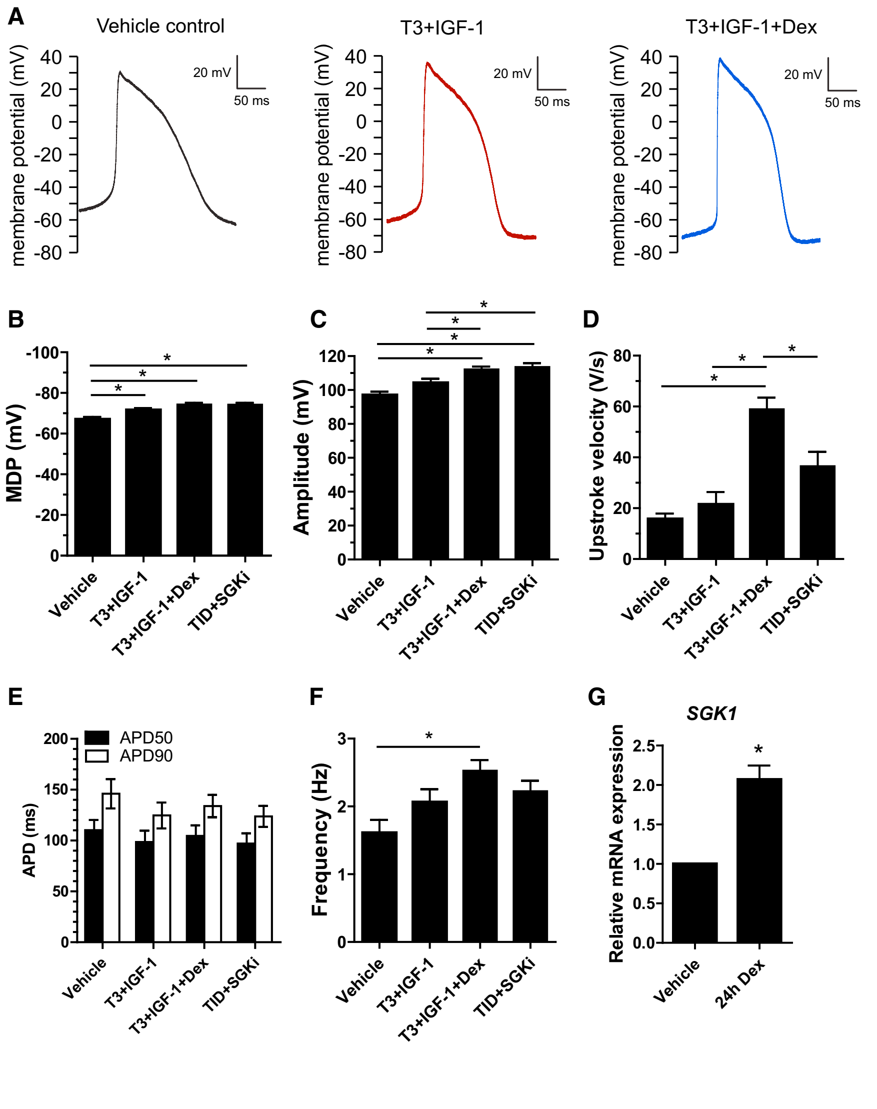

Figure 3. Cardiomyocyte Action Potential Measurement

(A) Typical examples of action potential (AP) traces from single spontaneously active cardiomyocytes maintained in the three conditions indicated.

(B–G) Average data of maximum diastolic potential (MDP), AP amplitude, AP upstroke velocity, and AP duration at 50% (APD50) and 90% (APD90) repolarization and AP frequency. SGKi, 5-day co-incubation with 6 μM GSK650394. Data are mean ± SEM. Actual AP values and n values are shown in Table 1. Statistical significance was calculated using a one-way ANOVA with Tukey's multiple comparison test *$p < 0.05$.

Procedures) showed a large increase (1.7-fold) in combined ATP turnover with TID (Figure 2D). The oligomycin-inhibited rates, the maximum uncoupled respiration rates induced by carbonyl cyanide-4-(trifluoromethoxy)phenylhydrazone (FCCP), and the non-mitochondrial rates were not significantly different between conditions (Figure 2B). These results support a synergistic effect for the actions of IGF-1 and Dex on basal cell activity. Inhibition of both excitation and contraction by co-injection of nifedipine and blebbistatin decreased the mitochondrial respiration rate by 39.3% ± 4.2% in the vehicle-only condition and 43.9% ± 9.0% in TID (Figure 1E) after subtraction of a minor buffer-only injection effect. As the starting respiration rate was higher with TID, this showed that the activity of these processes had increased at least proportionally by TID.

Sensitivity to substrate supply was assessed by examining the change in basal respiration rate and respiratory capacity on inhibition of either long-chain fatty acid uptake into mitochondria by the carnitine palmitoyl transferase inhibitor etomoxir (40 μM) or inhibition of mitochondrial pyruvate uptake by inhibition of the pyruvate transporter with UK5099 (5 μM). These experiments were performed in a more physiological medium containing 5.5 mM glucose, 0.15 mM sodium pyruvate, and 100 μM palmitate. Figures 2F and 2G show typical respiratory responses to etomoxir and UK5099 of cells from vehicle-only medium and TID medium, respectively. Figure 2H shows quantification of the sensitivity of basal and FCCP-stimulated mitochondrial respiration rates to these inhibitors. Basal respiration in cells from vehicle-only medium, and TID medium was inhibited 21.0% ± 4.3% and 24.4% ± 4.9%, respectively, by etomoxir, and 27.7% ± 4.2% and 31.9% ± 3.3%, respectively, by UK5099. The basal respiration rate was again higher in TID-treated cells. FCCP-stimulated respiration in cells from vehicle-only medium and TID medium was inhibited 18.5% ± 2.1% and 20.2% ± 2.2%, respectively, by etomoxir, and 53.8% ± 5.5% and 47.0% ± 2.9%, respectively, by UK5099. Overall, these data suggest that TID increases the basal utilization of both glucose and fatty acids in cardiomyocytes in concert with increased ATP demand for excitation/contraction as well as other processes. In line with these changes, TID increased expression of *PGC-1α* and *PGC-1β* (Figure S2), important regulators of myocardial fatty acid oxidation (FAO) and mitochondrial function (Birket et al., 2013; Finck and Kelly, 2007). We additionally found that increased bioenergetic activity by TID exposure was associated with a decreased rate of dihydroethidium (DHE) oxidation, suggesting a decreased level of reactive oxygen species (Figure S3), which may also support improved cardiomyocyte function.

Electrophysiological and Contractile Improvements by the Concerted Action of TID

To test whether the bioenergetic changes and improved resting ΔΨp resulted in improvements in electrophysiological function, action potentials were measured in spontaneously active cells. To focus on a possible effect of glucocorticoid signaling, we measured cells maintained in three conditions: vehicle-only, T3+IGF-1, and TID. The results are shown in Figure 3 and

Table 1. Action Potential Parameters of Single Spontaneously Active Cardiomyocytes

	Vehicle (n = 12)	T3+IGF-1 (n = 15)	T3+IGF-1+Dex (n = 33)	TID+SGKi (n = 16)
MDP (mV)	–66.9 ± 1.2	–71.5 ± 0.9[a]	–74.2 ± 0.8[a]	–74.2 ± 1.1[a]
dV/dt_{max} (V/s)	15.8 ± 2.2	21.5 ± 4.9	58.9 ± 4.6[a]	36.5 ± 5.6
APA (mV)	97 ± 2	104 ± 2[a]	112 ± 2[a]	114 ± 2[a]
APD_{50} (ms)	110 ± 11	98 ± 12	104 ± 11	97 ± 10
APD_{90} (ms)	146 ± 14	125 ± 13	134 ± 11	124 ± 10
Frequency (Hz)	1.6 ± 0.2	2.1 ± 0.2	2.5 ± 0.2[a]	2.2 ± 0.2
Capacitance (Cm)	20 ± 3	17 ± 1	25 ± 2	24 ± 3

Data are mean ± SEM; TID+SGKi = T3+IGF-1+Dex+GSK650394, n = number of cells; MDP, maximal diastolic potential; dV/dt_{max}, maximal upstroke velocity; APA, action potential amplitude; APD_{50} and APD_{90}, action potential duration at 50% and 90% repolarization, respectively.
[a]p < 0.05 compared to vehicle. One-way ANOVA with a Tukey post-test.

summarized in Table 1. The MDP was increased by both T3+IGF-1 and TID (Figure 3B), aligning well with our estimates by TMRM uptake. The amplitude of the action potential (AP) was also increased progressively by both treatments (Figure 3C). Interestingly, while the upstroke velocity was not significantly increased by T3+IGF-1 it was markedly increased by TID, from 16 ± 5 to 59 ± 6 V/s (Figure 3D; $p < 0.05$). This specific effect was significantly blocked by 6 μM GSK650394, an inhibitor of the serum- and glucocorticoid inducible kinase SGK1 (Sherk et al., 2008), which was previously shown to block cardiac sodium channel degradation, and which is activated by IGF-1 (Boehmer et al., 2003). We confirmed *SGK1* upregulation in response to Dex (Figure 3G). *SCN5A* expression was not increased by dexamethasone (Figure S2), and the inhibitor did not significantly affect any other aspect of the AP, together supporting this specific mechanistic explanation. Despite the increased MDP, AP frequency was increased by TID in this single-cell format (Figure 3F), differing from measurements in the monolayer format in Figure 2A. In summary, the AP data also support the conclusion that cardiomyocyte function was improved by the action of TID.

Next, we examined single-cell contractility under the same maintenance conditions with the exception that cardiomyocytes were plated on soft micropatterned polyacrylamide gels (20-μm-wide gelatin lines) containing fluorescent micro-beads. Bead displacement was imaged during spontaneous contraction of individual cells and converted to a value of traction stress (Figure 4A), as previously described (Ribeiro et al., 2015). In vehicle-only medium, mean traction stress was 0.15 ± 0.02 mN/mm^2; in T3+IGF-1, this was increased by 1.8-fold to 0.27 ± 0.02 mN/mm^2 ($p < 0.05$), and in TID medium it was increased by 2.9-fold to 0.44 ± 0.04 mN/mm^2 ($p < 0.05$) (Figure 4B). This corresponded to traction forces of 0.15 ± 0.02, 0.36 ± 0.04, and 0.76 ± 0.08 μN for vehicle-only, T3+IGF-1, and TID medium, respectively. The potentiated increase in traction stress by the addition of Dex, over the T3+IGF-1 condition, was significant ($p < 0.05$). Cardiomyocyte area was increased 1.4-fold by T3+IGF-1 ($p < 0.05$) and 1.8-fold by TID ($p < 0.05$) (Figure 4C), although cells remained small compared to adult human equivalents (Ancey et al., 2003). The potentiated increase in cell area by Dex was significant ($p < 0.05$), aligning with our estimates by eGFP intensity. Contraction frequency was increased by TID; however, as the force-frequency relationship was negative in all conditions, this did not explain the difference in traction stress between the conditions (Figure 4D). Co-staining of Troponin I and α-actinin showed that the structural organization of sarcomeres was improved by TID and sarcomeres were more uniform across the entire area of each cardiomyocyte (Figures 4E and S4). Expression of the contractility-related protein encoding genes *MYH6*, *ACTN2*, *MLC2V*, and *SERCA2* were upregulated by TID, although they were not significantly different between T3+IGF-1 and TID (Figure S2). T3 repressed *MYH7* expression as previously reported (Iwaki et al., 2014). An increase in traction stress by TID was also recorded in cardiomyocytes from the M1 hESC line (Figure S4).

Contractile Dysfunction in a hiPSC Model of Hypertrophic Cardiomyopathy

We have previously observed that single hiPSC-derived cardiomyocytes also generate very low contractile forces when maintained under standard basal conditions (~0.2 mN/mm^2) (Ribeiro et al., 2015). This creates a very low level of sensitivity for detecting defects in function, as even the control cells may be functioning far from their full dynamic range. Therefore, we took the opportunity to apply the TID-containing medium to an hiPSC disease model in which the derivative cardiomyocytes carry a c.2373dupG mutation in *MYBPC3* (Dambrot et al., 2014). This mutation causes HCM in patients (Alders et al., 2003). The mutant *MYBPC3* hiPSC-CMs were compared to cardiomyocytes derived from two hiPSC lines generated from healthy controls. Cardiac functional data of the HCM patients are provided in Table S1. A significant decrease in cMyBP-C protein relative to α-actinin was observed in the cardiomyocytes from all three HCM lines compared to both controls when measured at day 25 of differentiation (Figures 1A and 1B).

Attempts to measure these hiPSC-CMs in medium without TID proved unsuccessful as even the control cells failed to reliably generate robust bead displacement. However, in medium containing TID, traction force on the polyacrylamide substrate could be measured in all cell populations. Under these conditions, traction stress was significantly decreased in all three mutant lines, HCM1 0.31 ± 0.02, HCM2 0.30 ± 0.03, HCM3 0.29 ± 0.03 mN/mm^2 in comparison to both controls, Con1 0.57 ± 0.04 and Con2 0.51 ± 0.03 mN/mm^2; $p < 0.05$ (Figures 5C and 5D). This corresponded to traction forces of 0.43 ± 0.04, 0.46 ± 0.06, 0.44 ± 0.05, 0.81 ± 0.16, and 0.86 ± 0.10 μN for HCM1, HCM2, HCM3, Con1, and Con2, respectively. A difference in cardiomyocyte size as measured by cell area was not evident between the control and the mutant cells, suggesting an overt hypertrophic response had not occurred by this stage of development under these conditions (Figure 5E). Contraction frequencies were not different (Figure S4).

Reports have suggested that the HCM phenotype is promoted not by the mutated protein, which is not found in patients and was not detected in the current cell lysates, but by a decrease in the total level of wild-type cMyBP-C (haploinsufficiency) (van

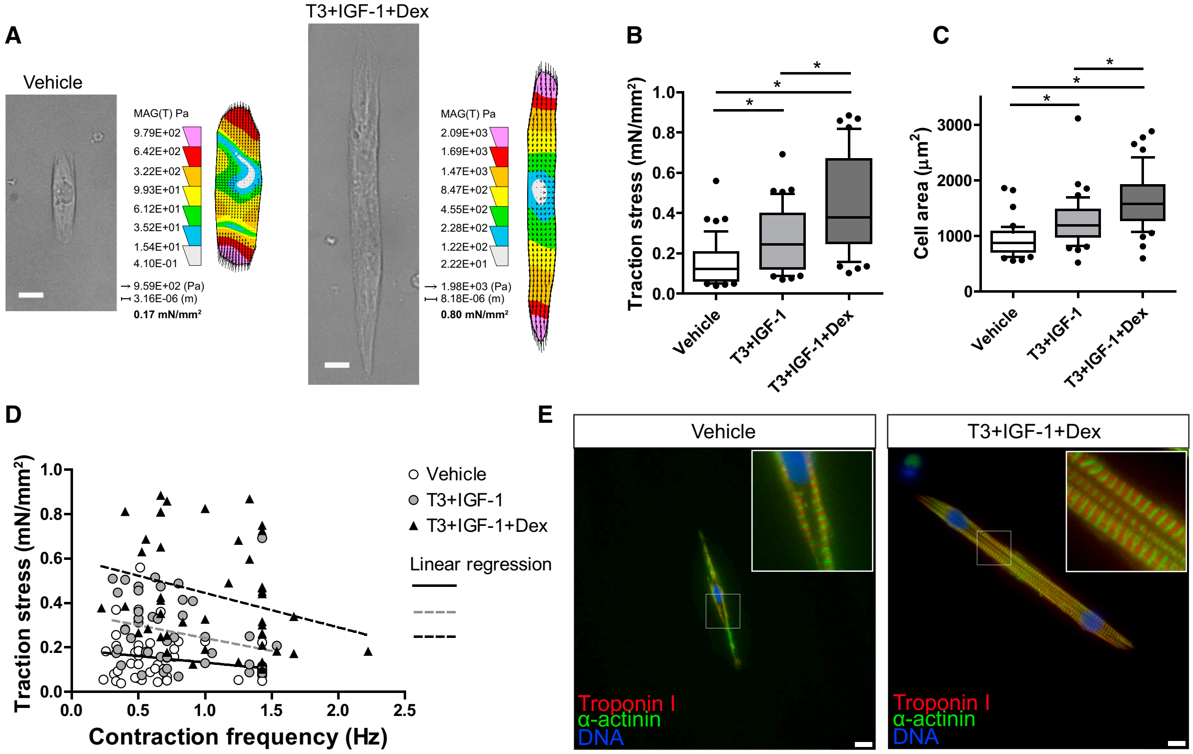

Figure 4. Single Cardiomyocyte Traction Force Measurement

(A) Typical examples of single aligned (spontaneously contracting) cardiomyocytes from vehicle-only and T3+IGF-1+Dex-containing medium, showing a bright-field image of the relaxed form and a heatmap of traction stress applied to the substrate calculated from the mean of the traction stress vectors (corresponding to Movie S1).

(B–D) (B) Traction stress, (C) cell area, and (D) traction stress-frequency relationship of single spontaneously contracting cardiomyocytes maintained in vehicle (n = 44), T3+IGF-1 (n = 44), and T3+IGF-1+Dex (n = 45).

(E) Immunostaining of typical aligned cardiomyocytes from vehicle- and TID-containing medium.

Box and whisker plots show the median, interquartile range, and 10–90 percentile range. The n signifies the number of individual cells measured, acquired over three independent experiments. Statistical significance was calculated using a one-way ANOVA with Tukey's multiple comparison test *$p < 0.05$. Scale bar, 10 μm. See also Figure S4.

Dijk et al., 2009; Moolman et al., 2000). To test whether the decrease in traction force was indeed caused by the decreased expression of cMyBP-C, we employed an RNAi approach in cardiomyocytes derived from the *NKX2-5$^{eGFP/w}$* hESC line. We transduced hESC-CMs with lentiviruses expressing scrambled or *MYBPC3*-specific short hairpin RNAs (shRNAs), selected them with puromycin, and assessed the effect on contractile force generation. Knockdown of cMyBP-C was confirmed at the protein level (Figure 5F). For these experiments, we used cardiomyocyte culture medium containing TID but further refined to contain entirely defined basal components and to be serum albumin free (see Experimental Procedures), these being important considerations for future use. Increased $NaHCO_3$ was also included to increase buffering of the high carbonic and lactic acid production of cardiomyocytes in TID. Dose-response tests were performed with T3 and Dex, and traction stress was compared to the previous formulation and found to be superior (Figure S5). Under this condition, traction stress was significantly decreased in cardiomyocytes expressing either of the *MYBPC3*-shRNA constructs (Scr 0.90 ± 0.05 mN/mm^2, shRNA1 0.47 ± 0.03 mN/mm^2, and shRNA2 0.36 ± 0.03 mN/mm^2; $p < 0.05$; corresponding to traction forces of 1.66 ± 0.11, 0.78 ± 0.07, and 0.70 ± 0.08 μN, respectively) (Figure 5G), while cell area was not significantly affected (Figure 5H), nor was protein content per cardiomyocyte as measured in bulk sorted populations (Scr shRNA: 185 ± 33 ng/1,000 cells versus *MYBPC3* shRNA 2: 189 ± 33 ng/1,000 cells). These results support the conclusion that the phenotype observed in the patient-derived cells is caused by the decreased level of cMyBP-C protein at a pre-hypertrophic stage.

DISCUSSION

Using a simple flow cytometry based assay, we have identified a combination of defined factors, namely, thyroid hormone (T3), IGF-1, and the glucocorticoid dexamethasone, which together enhanced the functional properties of hPSC-derived cardiomyocytes. We observed that T3 principally increased the resting membrane potential (ΔΨp), a critical factor in determining excitability and contractility, whereas IGF-1 and dexamethasone acted synergistically to stimulate cell energetics and traction force generation. In this optimized condition, we assessed the

OPEN ACCESS
CellPress

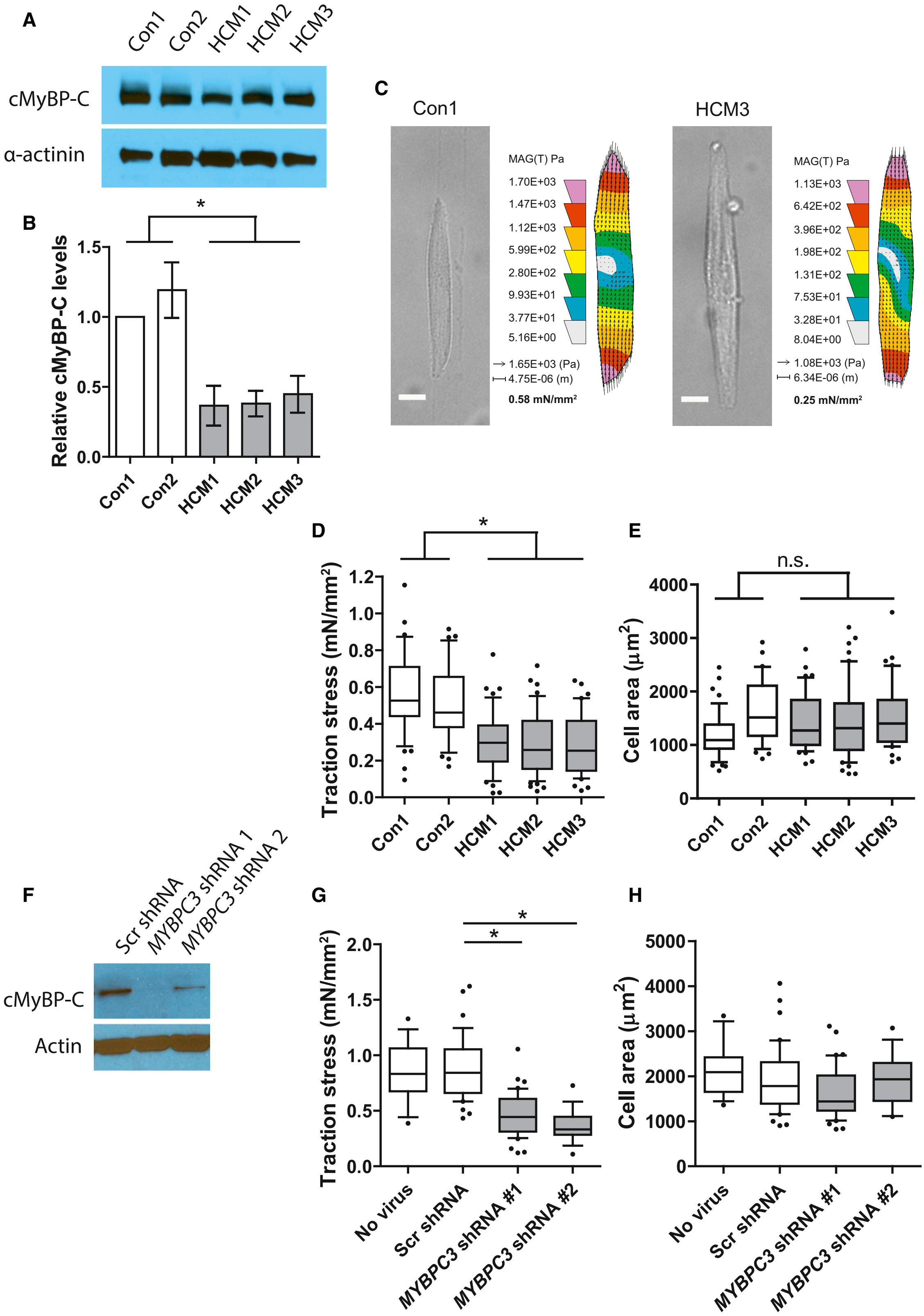

(legend on next page)

impact of an HCM-causing mutation in *MYBPC3,* or gene knockdown. Decreased force generation was observed in both cases, recapitulating measurements in mutant mouse models and patient-derived cells, opening up the possibility of using this in-vitro-based human model as a tool for future mechanistic studies and drug screening.

The positive influence of T3 on cardiomyocyte function is well supported in the literature. Levels increase markedly at birth in humans and have an important role in heart development and maturation (Biondi and Klein, 2004; Li et al., 2014). T3 was previously shown to increase the activity of the Na,K-ATPase in cardiomyocytes (Forini et al., 2004), as well as increasing the I_{k1} current (Sakaguchi et al., 1996), both of which could explain the increase in $\Delta\Psi p$ observed here. T3 also affects sarcomeric gene expression, stimulating expression of α-MHC, the fast ATPase activity MHC isoform, and repressing the expression of β-MHC, the slow ATPase activity isoform (Danzi et al., 2003; Iwaki et al., 2014). This effect was also shown in hESC-CMs by Yang et al. (2014) and confirmed here. These changes in MHC isoform expression may contribute to the increased contractile force generation observed with T3, as also previously reported by Yang et al. (2014). However, from this base condition we were able to extend these findings with the identification of additional physiologically relevant factors important for hPSC-CM function. IGF-1 also circulates in the developing heart and has an important role in myocardial cell growth and metabolism by signaling through the IGF-1 tyrosine kinase receptor (Troncoso et al., 2014). We found that IGF-1 mildly increased the size of hPSC-CMs, but more importantly proved an essential factor in revealing a positive functional role for the glucocorticoid dexamethasone in this system. Synergistic effects for IGF-1 and dexamethasone have been reported in skeletal muscle and heart, with pro-differentiation and anti-atrophic effects observed (Chrysis et al., 2011; Pansters et al., 2013; Schakman et al., 2008). Glucocorticoids also promote the structural and functional maturation of fetal mouse cardiomyocytes (Rog-Zielinska et al., 2013, 2015) but have little effect on hPSC-CMs when added alone although do affect calcium handling (G.K., C.L.M., M. Bellin, B. van Meer, L. Tertoolen, and S. Casini, unpublished data). Interestingly, the positive effect reported by Rog-Zielinska et al. in cultured cells was blocked by *PGC-1α* knockdown, a protein known to regulate mitochondrial respiration in cardiomyocytes (Birket et al., 2013). Our observation here, that in combination with IGF-1 dexamethasone exerted a clear bioenergetic response, also supports the conclusion that the functional improvements in force generation and electrophysiology may at least partly be the result of enhanced energy production pathways. Basal respiration coupled to ATP synthesis was increased by TID, and as this rate remained proportionally sensitive to inhibitors of excitation and contraction these processes must be more active in these cells. Additionally, we showed that basal respiration was sensitive to inhibitors of both mitochondrial pyruvate uptake as well as fatty acid uptake, implying that substrate supply also exerts significant respiratory control in both basal and TID conditions. The higher respiratory rate in TID suggests that both pyruvate and fatty acid usage was increased. TID also stimulated anaerobic glycolysis, which will provide additional pyruvate for mitochondria but can also drive the synthesis of biomass through precursor synthesis (Chen et al., 2015), and increase NADPH production important for ROS detoxification (Hamanaka and Chandel, 2011). Reduced DHE oxidation in cells treated with TID indeed indicated lower ROS levels. Mitochondrial ROS production may also be lower as a result of the increased ATP turnover (Goncalves et al., 2015). While ROS have been shown to have an important role in cardiomyocyte development (Puente et al., 2014), elevated levels can be inhibitory to cell function so a balance may be important (Birket et al., 2013; Guerra et al., 1996; Liu et al., 2010).

At the electrophysiological level, in the presence of T3, the addition of IGF-1 and dexamethasone caused a large increase in hESC-CM AP upstroke velocity not explained solely by the MDP. This was not associated with increased *SCN5A* expression and was therefore predicted to involve altered cardiac sodium channel regulation. The functional improvement was highly sensitive to small molecule inhibition of the glucocorticoid-inducible kinase SGK1 which is known to regulate degradation of the channel through inactivation of the ubiquitin ligase NEDD4-2 (Boehmer et al., 2003; Lang et al., 2014). This result supports a role for SGK1 in hPSC-CM function and will be an important avenue for future investigation. A further improvement was seen in the AP amplitude by TID, a parameter known to correlate with contractile force generation at a single-cell level

Figure 5. Single-Cell Traction Force Measurements in Cardiomyocytes with *MYBPC3* Mutation or *MYBPC3* shRNA Knockdown

(A) Western blot of cMyBP-C protein from two control (Con1 and Con2) and three *MYBPC3* mutation lines (HCM1, HCM2, and HCM3). α-actinin is shown as a loading control for cardiomyocyte input.

(B) Relative cMyBP-C levels normalized to α-actinin levels based on densitometry of western blot data (n = 3–6 lysates).

(C) Typical examples of single aligned (spontaneously contracting) cardiomyocytes from control (Con1) and *MYBPC3* mutation (HCM3) lines, showing a bright-field image of the relaxed form and a heatmap of traction stress applied to the substrate calculated from the mean of the traction stress vectors (corresponding to Movie S2).

(D and E) (D) Traction stress and (E) cell area of single spontaneously contracting iPSC-derived cardiomyocytes from two control (Con1 [n = 47] and Con2 [n = 36]) and three *MYBPC3* mutation lines (HCM1 [n = 44], HCM2 [n = 54], and HCM3 [n = 43]).

(F) Western blot of cMyBP-C protein in *NKX2-5$^{eGFP/w}$* hESC-derived cardiomyocytes after transduction with a scrambled (Scr) shRNA or two independent *MYBPC3*-specific shRNAs. Actin is shown as a loading control.

(G and H) (G) Traction stress and (H) cell area of single spontaneously contracting cardiomyocytes non-transduced (n = 15), stably expressing the Scr- (n = 32) or *MYBPC3*-specific (1, n = 26; 2, n = 16) shRNAs.

Boxplots and whisker plots show the median, interquartile range, and 10–90 percentile range. Unless otherwise stated, the n signifies the number of individual cells measured, acquired over three independent experiments. Statistical significance was tested with a one-way ANOVA with Tukey's multiple comparison test in (D) and (E), comparing either control against the HCM lines independently, and a Dunnett's correction in (G) and (H). Comparison to both controls in (D) are statistically significant *$p < 0.05$. Scale bar, 10 μm. See also Figure S5.

(Ribeiro et al., 2015). Indeed, TID substantially increased the traction force of hPSC-CMs as well as their sarcomeric structural organization, showing that all core aspects of cell function were enhanced.

Having identified conditions for maximizing traction force, we were able to apply the system to a cardiac disease model. HCM is caused by mutations in sarcomeric protein-encoding genes important for contractile function, of which mutation in *MYBPC3* accounts for approximately 20%–25% of cases. While animal models of cMyBP-C deficiency and patient-derived cells have been well studied (Barefield et al., 2015; van Dijk et al., 2009; Harris et al., 2002; McConnell et al., 2001; Pohlmann et al., 2007; Stöhr et al., 2013; Witjas-Paalberends et al., 2013), a widely accessible human in vitro model has been lacking. Here, using two independent approaches, we assessed the impact of cMyBP-C deficiency on hPSC-CM contractile force generation. Cardiomyocytes from patient-derived hiPSCs carrying a non-sense mutation in *MYBPC3* had <50% of normal cMyBP-C levels and were found to exert significantly less force at the single-cell level. This result was also observed following shRNA-mediated *MYBPC3* knockdown in otherwise normal cells. Our results are supported by measurements in adult patient-derived cells assessed in vitro where a 30%–40% decrease in maximum Ca^{2+}-activated force development has been observed in cells with the c.2373insG mutation, and a mechanism of haploinsufficiency suggested (van Dijk et al., 2009, 2014; Witjas-Paalberends et al., 2013). Importantly, the force generation defects we observed here were in non-hypertrophic cells, suggesting a primary event not consequential to hypertrophy and related maladaptive processes. This observation is consistent with the diastolic dysfunction reported in non-hypertrophic cardiomyocytes of mice heterozygous for a point mutation in *mybpc3* (Fraysse et al., 2012). Together these results support the possibility that the contractile dysfunction is an early initiating event in HCM disease pathogenesis. The specific initiating cause of the force generation defect in the hPSC-CMs studied here is unknown, as in all other models of *MYBPC3* mutation studied so far. Potential causes could include a defect in sarcomerogenesis leading to a reduced myofibril density, perturbed cross bridge cycling or increased sarcomere calcium sensitivity leading to diastolic dysfunction, or more general metabolic disturbance. Having a human model where this phenotype can be genetically induced and studied in a developmental context will present new opportunities to address this question. This could in turn lead to new therapeutic strategies for HCM, which is currently without cure.

In summary, providing functionally important physiological factors to hPSC-CMs may be critical for achieving robust baseline function and maximizing their use in applications of disease modeling, drug discovery and development, and toxicity screening. These are major goals for the stem cell field. Here, we found that three defined factors were sufficient to markedly improve the function of hPSC-CMs and facilitated their use in a disease model of HCM. The advance in culture conditions toward a fully defined formulation also revealed the potential of the single-cell traction force measurement technology for studying diseases of contractility in a highly controllable system.

EXPERIMENTAL PROCEDURES

hPSC Culture and Differentiation

H3 *NKX2-5*$^{eGFP/w}$ hESCs or M1 *NKX2-5*$^{eGFP/w}$ hESCs as previously generated (Elliott et al., 2011) were maintained on mouse embryonic fibroblasts and passaged using TrypLE select (Life Technologies). The generation of transgene-free hiPSCs from skin fibroblasts of one healthy male donor (LUMC0004iCtrl [Con1]) and three patients each with a c.2373dupG mutation in *MYBPC3* (LUMC0033iMyBPC [HCM1], LUMC0034iMyBPC [HCM2], and LUMC0035iMyBPC [HCM3]) was previously reported (Dambrot et al., 2014). A second transgene-free control hiPSC line (LUMC0047iCtrl [Con2]) generated from another healthy male donor was included in this study. hiPSCs were maintained on Matrigel (growth factor reduced; Corning 354230) in mTeSR1 medium (Stem Cell Technologies) and passaged with 1 mg/ml Dispase (Life Technologies). *NKX2-5*$^{eGFP/w}$ hiPSCs (R.P.D. and C.L.M., unpublished data) were maintained in Essential 8 medium (Life Technologies) and differentiated as previously described (van den Berg et al., 2015).

Cardiac differentiation was induced from monolayer cultures on Matrigel in a serum-free medium (BSA, polyvinyl alcohol, essential lipids [BPEL]) as described in the Supplemental Information. Contracting cultures were dissociated on day 13 and replated on Matrigel-coated 24-well plates. The following experimental factors were added on day 16, refreshed on day 20, and measured on day 21: 100 ng/ml Long R3 IGF-1 (in the main text: IGF-1), 1 μM SAG (Millipore), 1 μM dexamethasone, 100 nM triiodothyronine hormone, 10 μM phenylephrine, 1 μM isoproterenol, and 1-1000 nM norepinephrine. Unless otherwise stated, all factors were obtained from Sigma-Aldrich.

The composition of the defined cardiomyocyte medium used in the *MYBPC3* shRNA experiment can be found in the Supplemental Information.

Lentiviral Transduction

shRNAs against *MYBPC3* (NM_000256) were obtained from Open Biosystems in the pLKO vector (1, TRCN0000082906; 2, TRCN0000082903). A scrambled shRNA was used as control (Sarbassov et al., 2005) (Addgene plasmid: 1864). Cardiomyocytes were transduced with lentiviruses on day 15 of differentiation and subsequently selected with puromycin before single-cell dissociation and measurement. Protein knockdown was assessed by western blot as described in the Supplemental Information.

Flow Cytometry Measurements for Plasma Membrane Potential and Reactive Oxygen Species

For relative plasma membrane potential measurement, differentiated cultures were dissociated on day 21 (after treatment as above from day 16) using 5× TrypLE Select and resuspended in 2.5 nM TMRM (Life Technologies) in warm assay medium. Cells were incubated for 8 min at 37°C before being measured by flow cytometry. For superoxide detection, dissociated cells were labeled with 5 μM DHE for 30 min at 37°C and then measured by flow cytometry. Appropriate compensation to correct from cross-bleed was performed for each.

Respiration and Acidification Rates Measured with the Seahorse XF Analyzer

Respiration and acidification rates were measured using a Seahorse XF-24 or an XF-96 Analyzer (Seahorse Bioscience). Cells were seeded on Matrigel-coated assay plates 7 days before measurement. The assay was performed in bicarbonate-free DMEM as described in the Supplemental Information. Cells were washed twice and pre-incubated in the assay medium for 1 hr before measurement. For the standard profiling, oligomycin was used at 0.5 μg/ml, FCCP titrated in two injections to 3 μM, and rotenone and antimycin A were added at 1 and 2 μM, respectively. A standard protein assay was used to normalize values to whole-cell protein. Glycolytic rate calculations and ATP production rates are described in the Supplemental Information. To assess ATP demand for excitation and contractility, nifedipine (10 μM) and blebbistatin (5 μg/ml) were co-injected, and the respiration rate was immediately recorded, followed by measurements after oligomycin and then rotenone and antimycin A injection. A vehicle-only injection was performed in parallel and the effect was subtracted. Experiments analyzing the effect of etomoxir and UK5099 were performed on the XF-96 format using an assay medium

with the following modifications: the glucose concentration was 5.5 mM, sodium pyruvate 0.15 mM, and palmitic acid was included at 100 μM conjugated to fatty-acid-free BSA as described in the Supplemental Information. 10 mM HEPES was also included in this assay medium to provide extra buffering (glycolytic rates were not calculated). Cells were pre-incubated in this assay medium for 4 hr before measurement. To assess the response to substrate uptake inhibitors, 40 μM etomoxir or 5 μM UK5099 was injected, and respiration rates were recorded after 45 min, followed by injections of oligomycin, FCCP, and then rotenone and antimycin A. A vehicle-only injection was performed in parallel, and the effect was subtracted.

Electrophysiological Characterization

Action potential (AP) recordings were performed on single cardiomyocytes, 6–10 days after cell dissociation with the amphotericin perforated patch-clamp technique using an Axopatch 200B amplifier (Molecular Devices Corporation). Signals were filtered and digitized at 5 and 40 kHz, respectively. Data acquisition and analysis were accomplished using pClamp10.1 (Axon Instruments) and custom software. Potentials were corrected for the liquid junction potential. Cells were continuously perfused in a perfusion chamber at 37°C (Cell MicroControls) using Tyrode's solution containing (mM) NaCl 140, KCl 5.4, $CaCl_2$ 1.8, $MgCl_2$ 1.0, glucose 5.5, HEPES 5 (pH 7.4) (NaOH). Pipettes (borosilicate glass; resistance ~2.5 MΩ) were filled with solution containing (mM) K-gluconate 125, KCl 20, NaCl 5, amphotericin-B 0.22, HEPES 10 (pH 7.2) (KOH).

APs were recorded at spontaneous frequencies and characterized by duration at 50% and 90% repolarization (APD50 and APD90, respectively), maximal diastolic potential (MDP), AP amplitude, maximal upstroke velocity, and frequency. AP parameter values obtained from eight to nine consecutive APs were averaged, and data were collected from at least two independent differentiations per condition.

The SGK1 inhibitor GSK650394 (Sherk et al., 2008) or vehicle-only control was applied at 6 μM 5 days before AP measurement. AP measurements were blinded in acquisition and analysis.

Traction Force Measurements

The traction force measurements were performed as previously described (Hazeltine et al., 2012). Cells were seeded on gelatine patterned acrylamide gels (see the Supplemental Information) 4 days before and measured in their normal culture medium in an environment at 37°C with 5% CO_2. An image series of aligned single spontaneously contracting cardiomyocytes was taken at 40× magnification at 20 frames per second, recording bright-field and fluorescent beads. Single frames from maximal relaxation and contraction of the bright-field and fluorescent beads image-series were analyzed by the LIBTRC software package (kindly provided by Dr. Micah Dembo), creating a mask of the cell outline from the bright-field image and a vector map from the difference between the relaxed and contracted fluorescent beads images. The vector map and the cell mask were used to calculate the maximum total force that the cell applies on the substrate. The traction stress generated by the cardiomyocyte during contraction was calculated by dividing the total force by the cell-surface area. Measurements were blinded in acquisition and analysis.

Quantitative Real-Time PCR

RNA was isolated with a Minelute RNA extraction kit (QIAGEN) and cDNA synthesized using an iScript cDNA synthesis kit (Bio-Rad). Real-time PCR was performed on a Bio-Rad CFX384 machine using IQ SYBR Green (Bio-Rad). Gene expression values were normalized to the mean expression of the housekeeping genes human ribosomal protein (*RPLP0*), glucuronidase (*GUSB*), and *RNF7*. Primer sequences can be found in the Supplemental Information.

SUPPLEMENTAL INFORMATION

Supplemental Information includes Supplemental Experimental Procedures, five figures, one table, and two movies and can be found with this article online at http://dx.doi.org/10.1016/j.celrep.2015.09.025.

AUTHOR CONTRIBUTIONS

Conceptualization, M.J.B., M.C.R., R.P., and C.L.M.; Investigation, M.J.B., M.C.R., G.K., V.v.d.P., A.R.L., H.D.D., R.P.D., and D.W.; Writing – Original Draft, M.J.B. and C.L.M.; Writing – Review & Editing, M.J.B., M.C.R., P.G.M., R.P., and C.L.M.; Funding Acquisition, C.L.M., R.P., and P.G.M.; Resources, C.D., D.E.A., P.G.M.; Supervision, C.L.M. and R.P.

ACKNOWLEDGMENTS

Work is supported by Cardiovascular Research Netherlands (CVON HUSTCARE), Netherlands Institute of Regenerative Medicine (NIRM), the European Research Council (ERCAdG 323182 STEMCARDIOVASC), the Rembrandt Institute of Cardiovascular Science RICS, and the Netherlands Organization for Scientific Research (NWO-FOM FOM 09MMC02). Other support includes ZonMw-MKMD-40-42600-98-036 (R.P.), Netherlands Genomics Initiative NGI/NWO 05040202 (P.M.), and Marie Curie IRG 247918 (P.M.). The Seahorse Extracellular Flux Analyzer was purchased through the generous contribution of the Dorpmans-Wigmans Stichting. We thank Dr. Christian Freund and Simona van de Pas of the LUMC hiPSC Core Facility for the "Con1" hiPSC line, Dr. Sakthivel Sadayappan (Loyola University Chicago) for the cMyBP-C antibody, and Ajit Divakaruni (University of California, San Diego) for helpful advice with the design of Seahorse experiments.

Received: May 6, 2015
Revised: July 31, 2015
Accepted: September 5, 2015
Published: October 15, 2015

REFERENCES

Alders, M., Jongbloed, R., Deelen, W., van den Wijngaard, A., Doevendans, P., Ten Cate, F., Regitz-Zagrosek, V., Vosberg, H.P., van Langen, I., Wilde, A., et al. (2003). The 2373insG mutation in the MYBPC3 gene is a founder mutation, which accounts for nearly one-fourth of the HCM cases in the Netherlands. Eur. Heart J. *24*, 1848–1853.

Ancey, C., Menet, E., Corbi, P., Fredj, S., Garcia, M., Rücker-Martin, C., Bescond, J., Morel, F., Wijdenes, J., Lecron, J.-C., and Potreau, D. (2003). Human cardiomyocyte hypertrophy induced in vitro by gp130 stimulation. Cardiovasc. Res. *59*, 78–85.

Barefield, D., Kumar, M., Gorham, J., Seidman, J.G., Seidman, C.E., de Tombe, P.P., and Sadayappan, S. (2015). Haploinsufficiency of MYBPC3 exacerbates the development of hypertrophic cardiomyopathy in heterozygous mice. J. Mol. Cell. Cardiol. *79*, 234–243.

Bellin, M., Casini, S., Davis, R.P., D'Aniello, C., Haas, J., Ward-van Oostwaard, D., Tertoolen, L.G.J., Jung, C.B., Elliott, D.A., Welling, A., et al. (2013). Isogenic human pluripotent stem cell pairs reveal the role of a KCNH2 mutation in long-QT syndrome. EMBO J. *32*, 3161–3175.

Biondi, B., and Klein, I. (2004). Hypothyroidism as a risk factor for cardiovascular disease. Endocrine *24*, 1–13.

Birket, M.J., Casini, S., Kosmidis, G., Elliott, D.A., Gerencser, A.A., Baartscheer, A., Schumacher, C., Mastroberardino, P.G., Elefanty, A.G., Stanley, E.G., and Mummery, C.L. (2013). PGC-1α and reactive oxygen species regulate human embryonic stem cell-derived cardiomyocyte function. Stem Cell Reports *1*, 560–574.

Birket, M.J., Ribeiro, M.C., Verkerk, A.O., Ward, D., Leitoguinho, A.R., den Hartogh, S.C., Orlova, V.V., Devalla, H.D., Schwach, V., Bellin, M., et al. (2015). Expansion and patterning of cardiovascular progenitors derived from human pluripotent stem cells. Nat. Biotechnol. *33*, 970–979. http://dx.doi.org/10.1038/nbt.3271.

Bisping, E., Ikeda, S., Sedej, M., Wakula, P., McMullen, J.R., Tarnavski, O., Sedej, S., Izumo, S., Pu, W.T., and Pieske, B. (2012). Transcription factor GATA4 is activated but not required for insulin-like growth factor 1 (IGF1)-induced cardiac hypertrophy. J. Biol. Chem. *287*, 9827–9834.

Boehmer, C., Wilhelm, V., Palmada, M., Wallisch, S., Henke, G., Brinkmeier, H., Cohen, P., Pieske, B., and Lang, F. (2003). Serum and glucocorticoid inducible kinases in the regulation of the cardiac sodium channel SCN5A. Cardiovasc. Res. *57*, 1079–1084.

Chen, X., Qian, Y., and Wu, S. (2015). The Warburg effect: evolving interpretations of an established concept. Free Radic. Biol. Med. *79*, 253–263.

Chrysis, D., Chagin, A., and Sävendahl, L. (2011). Insulin-like growth factor-1 restores dexamethasone-induced heart growth arrest in rats: the role of the ubiquitin pathway. Hormones (Athens) *10*, 46–56.

Clement, C.A., Kristensen, S.G., Møllgård, K., Pazour, G.J., Yoder, B.K., Larsen, L.A., and Christensen, S.T. (2009). The primary cilium coordinates early cardiogenesis and hedgehog signaling in cardiomyocyte differentiation. J. Cell Sci. *122*, 3070–3082.

Dambrot, C., Braam, S.R., Tertoolen, L.G.J., Birket, M., Atsma, D.E., and Mummery, C.L. (2014). Serum supplemented culture medium masks hypertrophic phenotypes in human pluripotent stem cell derived cardiomyocytes. J. Cell. Mol. Med. *18*, 1509–1518.

Danzi, S., Ojamaa, K., and Klein, I. (2003). Triiodothyronine-mediated myosin heavy chain gene transcription in the heart. Am. J. Physiol. Heart Circ. Physiol. *284*, H2255–H2262.

Drawnel, F.M., Boccardo, S., Prummer, M., Delobel, F., Graff, A., Weber, M., Gérard, R., Badi, L., Kam-Thong, T., Bu, L., et al. (2014). Disease modeling and phenotypic drug screening for diabetic cardiomyopathy using human induced pluripotent stem cells. Cell Rep. *9*, 810–821.

El-Armouche, A., and Eschenhagen, T. (2009). Beta-adrenergic stimulation and myocardial function in the failing heart. Heart Fail. Rev. *14*, 225–241.

Elliott, D.A., Braam, S.R., Koutsis, K., Ng, E.S., Jenny, R., Lagerqvist, E.L., Biben, C., Hatzistavrou, T., Hirst, C.E., Yu, Q.C., et al. (2011). NKX2-5(eGFP/w) hESCs for isolation of human cardiac progenitors and cardiomyocytes. Nat. Methods *8*, 1037–1040.

Eschenhagen, T., Mummery, C., and Knollmann, B.C. (2015). Modelling sarcomeric cardiomyopathies in the dish: from human heart samples to iPSC cardiomyocytes. Cardiovasc. Res. *105*, 424–438.

Finck, B.N., and Kelly, D.P. (2007). Peroxisome proliferator-activated receptor gamma coactivator-1 (PGC-1) regulatory cascade in cardiac physiology and disease. Circulation *115*, 2540–2548.

Földes, G., Matsa, E., Kriston-Vizi, J., Leja, T., Amisten, S., Kolker, L., Kodagoda, T., Dolatshad, N.F., Mioulane, M., Vauchez, K., et al. (2014). Aberrant α-adrenergic hypertrophic response in cardiomyocytes from human induced pluripotent cells. Stem Cell Reports *3*, 905–914.

Forini, F., Nicolini, G., Balzan, S., Ratto, G.M., Murzi, B., Vanini, V., and Iervasi, G. (2004). Amiodarone inhibits the 3,5,3′-triiodothyronine-dependent increase of sodium/potassium adenosine triphosphatase activity and concentration in human atrial myocardial tissue. Thyroid *14*, 493–499.

Fraysse, B., Weinberger, F., Bardswell, S.C., Cuello, F., Vignier, N., Geertz, B., Starbatty, J., Krämer, E., Coirault, C., Eschenhagen, T., et al. (2012). Increased myofilament Ca2+ sensitivity and diastolic dysfunction as early consequences of Mybpc3 mutation in heterozygous knock-in mice. J. Mol. Cell. Cardiol. *52*, 1299–1307.

Gerencser, A.A., Chinopoulos, C., Birket, M.J., Jastroch, M., Vitelli, C., Nicholls, D.G., and Brand, M.D. (2012). Quantitative measurement of mitochondrial membrane potential in cultured cells: calcium-induced de- and hyperpolarization of neuronal mitochondria. J. Physiol. *590*, 2845–2871.

Goncalves, R.L.S., Quinlan, C.L., Perevoshchikova, I.V., Hey-Mogensen, M., and Brand, M.D. (2015). Sites of superoxide and hydrogen peroxide production by muscle mitochondria assessed ex vivo under conditions mimicking rest and exercise. J. Biol. Chem. *290*, 209–227.

Guerra, L., Cerbai, E., Gessi, S., Borea, P.A., and Mugelli, A. (1996). The effect of oxygen free radicals on calcium current and dihydropyridine binding sites in guinea-pig ventricular myocytes. Br. J. Pharmacol. *118*, 1278–1284.

Hamanaka, R.B., and Chandel, N.S. (2011). Cell biology. Warburg effect and redox balance. Science *334*, 1219–1220.

Han, L., Li, Y., Tchao, J., Kaplan, A.D., Lin, B., Li, Y., Mich-Basso, J., Lis, A., Hassan, N., London, B., et al. (2014). Study familial hypertrophic cardiomyopathy using patient-specific induced pluripotent stem cells. Cardiovasc. Res. *104*, 258–269.

Harris, S.P., Bartley, C.R., Hacker, T.A., McDonald, K.S., Douglas, P.S., Greaser, M.L., Powers, P.A., and Moss, R.L. (2002). Hypertrophic cardiomyopathy in cardiac myosin binding protein-C knockout mice. Circ. Res. *90*, 594–601.

Hazeltine, L.B., Simmons, C.S., Salick, M.R., Lian, X., Badur, M.G., Han, W., Delgado, S.M., Wakatsuki, T., Crone, W.C., Pruitt, B.L., and Palecek, S.P. (2012). Effects of substrate mechanics on contractility of cardiomyocytes generated from human pluripotent stem cells. Int. J. Cell Biol. *2012*, 508294.

Iwaki, H., Sasaki, S., Matsushita, A., Ohba, K., Matsunaga, H., Misawa, H., Oki, Y., Ishizuka, K., Nakamura, H., and Suda, T. (2014). Essential role of TEA domain transcription factors in the negative regulation of the MYH 7 gene by thyroid hormone and its receptors. PLoS ONE *9*, e88610.

Lan, F., Lee, A.S., Liang, P., Sanchez-Freire, V., Nguyen, P.K., Wang, L., Han, L., Yen, M., Wang, Y., Sun, N., et al. (2013). Abnormal calcium handling properties underlie familial hypertrophic cardiomyopathy pathology in patient-specific induced pluripotent stem cells. Cell Stem Cell *12*, 101–113.

Lang, F., Stournaras, C., and Alesutan, I. (2014). Regulation of transport across cell membranes by the serum- and glucocorticoid-inducible kinase SGK1. Mol. Membr. Biol. *31*, 29–36.

Li, M., Iismaa, S.E., Naqvi, N., Nicks, A., Husain, A., and Graham, R.M. (2014). Thyroid hormone action in postnatal heart development. Stem Cell Res. (Amst.) *13* (*3 Pt B*), 582–591.

Liu, M., Liu, H., and Dudley, S.C., Jr. (2010). Reactive oxygen species originating from mitochondria regulate the cardiac sodium channel. Circ. Res. *107*, 967–974.

Ma, J., Guo, L., Fiene, S.J., Anson, B.D., Thomson, J.A., Kamp, T.J., Kolaja, K.L., Swanson, B.J., and January, C.T. (2011). High purity human-induced pluripotent stem cell-derived cardiomyocytes: electrophysiological properties of action potentials and ionic currents. Am. J. Physiol. Heart Circ. Physiol. *301*, H2006–H2017.

Magyar, J., Iost, N., Körtvély, A., Bányász, T., Virág, L., Szigligeti, P., Varró, A., Opincariu, M., Szécsi, J., Papp, J.G., and Nánási, P.P. (2000). Effects of endothelin-1 on calcium and potassium currents in undiseased human ventricular myocytes. Pflugers Arch. *441*, 144–149.

Maron, B.J., and Maron, M.S. (2013). Hypertrophic cardiomyopathy. Lancet *381*, 242–255.

McConnell, B.K., Fatkin, D., Semsarian, C., Jones, K.A., Georgakopoulos, D., Maguire, C.T., Healey, M.J., Mudd, J.O., Moskowitz, I.P.G., Conner, D.A., et al. (2001). Comparison of two murine models of familial hypertrophic cardiomyopathy. Circ. Res. *88*, 383–389.

Mookerjee, S.A., Goncalves, R.L.S., Gerencser, A.A., Nicholls, D.G., and Brand, M.D. (2015). The contributions of respiration and glycolysis to extracellular acid production. Biochim. Biophys. Acta *1847*, 171–181.

Moolman, J.A., Reith, S., Uhl, K., Bailey, S., Gautel, M., Jeschke, B., Fischer, C., Ochs, J., McKenna, W.J., Klues, H., and Vosberg, H.P. (2000). A newly created splice donor site in exon 25 of the MyBP-C gene is responsible for inherited hypertrophic cardiomyopathy with incomplete disease penetrance. Circulation *101*, 1396–1402.

Ng, E.S., Davis, R., Stanley, E.G., and Elefanty, A.G. (2008). A protocol describing the use of a recombinant protein-based, animal product-free medium (APEL) for human embryonic stem cell differentiation as spin embryoid bodies. Nat. Protoc. *3*, 768–776.

Pansters, N.A., Langen, R.C., Wouters, E.F., and Schols, A.M. (2013). Synergistic stimulation of myogenesis by glucocorticoid and IGF-I signaling. J. Appl. Physiol. *114*, 1329–1339.

Pohlmann, L., Kröger, I., Vignier, N., Schlossarek, S., Krämer, E., Coirault, C., Sultan, K.R., El-Armouche, A., Winegrad, S., Eschenhagen, T., and Carrier, L. (2007). Cardiac myosin-binding protein C is required for complete relaxation in intact myocytes. Circ. Res. *101*, 928–938.

Puente, B.N., Kimura, W., Muralidhar, S.A., Moon, J., Amatruda, J.F., Phelps, K.L., Grinsfelder, D., Rothermel, B.A., Chen, R., Garcia, J.A., et al. (2014). The oxygen-rich postnatal environment induces cardiomyocyte cell-cycle arrest through DNA damage response. Cell *157*, 565–579.

Ribeiro, M.C., Tertoolen, L.G., Guadix, J.A., Bellin, M., Kosmidis, G., D'Aniello, C., Monshouwer-Kloots, J., Goumans, M.-J., Wang, Y.L., Feinberg, A.W., et al. (2015). Functional maturation of human pluripotent stem cell derived cardiomyocytes in vitro–correlation between contraction force and electrophysiology. Biomaterials *51*, 138–150.

Rog-Zielinska, E.A., Thomson, A., Kenyon, C.J., Brownstein, D.G., Moran, C.M., Szumska, D., Michailidou, Z., Richardson, J., Owen, E., Watt, A., et al. (2013). Glucocorticoid receptor is required for foetal heart maturation. Hum. Mol. Genet. *22*, 3269–3282.

Rog-Zielinska, E.A., Craig, M.-A., Manning, J.R., Richardson, R.V., Gowans, G.J., Dunbar, D.R., Gharbi, K., Kenyon, C.J., Holmes, M.C., Hardie, D.G., et al. (2015). Glucocorticoids promote structural and functional maturation of foetal cardiomyocytes: a role for PGC-1α. Cell Death Differ. *22*, 1106–1116.

Sakaguchi, Y., Cui, G., and Sen, L. (1996). Acute effects of thyroid hormone on inward rectifier potassium channel currents in guinea pig ventricular myocytes. Endocrinology *137*, 4744–4751.

Sarbassov, D.D., Guertin, D.A., Ali, S.M., and Sabatini, D.M. (2005). Phosphorylation and regulation of Akt/PKB by the rictor-mTOR complex. Science *307*, 1098–1101.

Sartiani, L., Bettiol, E., Stillitano, F., Mugelli, A., Cerbai, E., and Jaconi, M.E. (2007). Developmental changes in cardiomyocytes differentiated from human embryonic stem cells: a molecular and electrophysiological approach. Stem Cells *25*, 1136–1144.

Schakman, O., Kalista, S., Bertrand, L., Lause, P., Verniers, J., Ketelslegers, J.M., and Thissen, J.P. (2008). Role of Akt/GSK-3beta/beta-catenin transduction pathway in the muscle anti-atrophy action of insulin-like growth factor-I in glucocorticoid-treated rats. Endocrinology *149*, 3900–3908.

Sherk, A.B., Frigo, D.E., Schnackenberg, C.G., Bray, J.D., Laping, N.J., Trizna, W., Hammond, M., Patterson, J.R., Thompson, S.K., Kazmin, D., et al. (2008). Development of a small-molecule serum- and glucocorticoid-regulated kinase-1 antagonist and its evaluation as a prostate cancer therapeutic. Cancer Res. *68*, 7475–7483.

Stöhr, A., Friedrich, F.W., Flenner, F., Geertz, B., Eder, A., Schaaf, S., Hirt, M.N., Uebeler, J., Schlossarek, S., Carrier, L., et al. (2013). Contractile abnormalities and altered drug response in engineered heart tissue from Mybpc3-targeted knock-in mice. J. Mol. Cell. Cardiol. *63*, 189–198.

Synnergren, J., Améen, C., Jansson, A., and Sartipy, P. (2012). Global transcriptional profiling reveals similarities and differences between human stem cell-derived cardiomyocyte clusters and heart tissue. Physiol. Genomics *44*, 245–258.

Troncoso, R., Ibarra, C., Vicencio, J.M., Jaimovich, E., and Lavandero, S. (2014). New insights into IGF-1 signaling in the heart. Trends Endocrinol. Metab. *25*, 128–137.

van den Berg, C.W., Elliott, D.A., Braam, S.R., Mummery, C.L., and Davis, R.P. (2015). Differentiation of human pluripotent stem cells to cardiomyocytes under defined conditions. Methods Mol. Biol. http://dx.doi.org/10.1007/7651_2014_178

van Dijk, S.J., Dooijes, D., dos Remedios, C., Michels, M., Lamers, J.M.J., Winegrad, S., Schlossarek, S., Carrier, L., ten Cate, F.J., Stienen, G.J.M., and van der Velden, J. (2009). Cardiac myosin-binding protein C mutations and hypertrophic cardiomyopathy: haploinsufficiency, deranged phosphorylation, and cardiomyocyte dysfunction. Circulation *119*, 1473–1483.

van Dijk, S.J., Boontje, N.M., Heymans, M.W., Ten Cate, F.J., Michels, M., Dos Remedios, C., Dooijes, D., van Slegtenhorst, M.A., van der Velden, J., and Stienen, G.J.M. (2014). Preserved cross-bridge kinetics in human hypertrophic cardiomyopathy patients with MYBPC3 mutations. Pflugers Arch. *466*, 1619–1633.

Veerman, C.C., Kosmidis, G., Mummery, C.L., Casini, S., Verkerk, A.O., and Bellin, M. (2015). Immaturity of human stem-cell-derived cardiomyocytes in culture: fatal flaw or soluble problem? Stem Cells Dev. *24*, 1035–1052.

Vidal, M., Wieland, T., Lohse, M.J., and Lorenz, K. (2012). β-Adrenergic receptor stimulation causes cardiac hypertrophy via a Gβγ/Erk-dependent pathway. Cardiovasc. Res. *96*, 255–264.

Wang, G., McCain, M.L., Yang, L., He, A., Pasqualini, F.S., Agarwal, A., Yuan, H., Jiang, D., Zhang, D., Zangi, L., et al. (2014). Modeling the mitochondrial cardiomyopathy of Barth syndrome with induced pluripotent stem cell and heart-on-chip technologies. Nat. Med. *20*, 616–623.

Witjas-Paalberends, E.R., Piroddi, N., Stam, K., van Dijk, S.J., Oliviera, V.S., Ferrara, C., Scellini, B., Hazebroek, M., ten Cate, F.J., van Slegtenhorst, M., et al. (2013). Mutations in MYH7 reduce the force generating capacity of sarcomeres in human familial hypertrophic cardiomyopathy. Cardiovasc. Res. *99*, 432–441.

Yang, X., Rodriguez, M., Pabon, L., Fischer, K.A., Reinecke, H., Regnier, M., Sniadecki, N.J., Ruohola-Baker, H., and Murry, C.E. (2014). Tri-iodo-l-thyronine promotes the maturation of human cardiomyocytes-derived from induced pluripotent stem cells. J. Mol. Cell. Cardiol. *72*, 296–304.

Zhang, J., Wilson, G.F., Soerens, A.G., Koonce, C.H., Yu, J., Palecek, S.P., Thomson, J.A., and Kamp, T.J. (2009). Functional cardiomyocytes derived from human induced pluripotent stem cells. Circ. Res. *104*, e30–e41.

Zhang, Z., Liu, R., Townsend, P.A., and Proud, C.G. (2013). p90(RSK)s mediate the activation of ribosomal RNA synthesis by the hypertrophic agonist phenylephrine in adult cardiomyocytes. J. Mol. Cell. Cardiol. *59*, 139–147.

Zhang, M., D'Aniello, C., Verkerk, A.O., Wrobel, E., Frank, S., Ward-van Oostwaard, D., Piccini, I., Freund, C., Rao, J., Seebohm, G., et al. (2014). Recessive cardiac phenotypes in induced pluripotent stem cell models of Jervell and Lange-Nielsen syndrome: disease mechanisms and pharmacological rescue. Proc. Natl. Acad. Sci. USA *111*, E5383–E5392.

Cell Reports
Supplemental Information

Contractile Defect Caused by Mutation in *MYBPC3* Revealed under Conditions Optimized for Human PSC-Cardiomyocyte Function

Matthew J. Birket, Marcelo C. Ribeiro, Georgios Kosmidis, Dorien Ward, Ana Rita Leitoguinho, Vera van de Pol, Cheryl Dambrot, Harsha D. Devalla, Richard P. Davis, Pier G. Mastroberardino, Douwe E. Atsma, Robert Passier, and Christine L. Mummery

Inventory of Supplementary Information for:

"Contractile defect caused by mutation in *MYBPC3* revealed under conditions optimized for human PSC-cardiomyocyte function"

Correspondence to: Christine L Mummery PhD, Leiden University Medical Center, 2300 RC Leiden, The Netherlands. Telephone: +31-71-526 9307 Fax: +31-71-526 8289 Email: c.l.mummery@lumc.nl

- **Figure S1**
- **Figure S2**
- **Figure S3**
- **Figure S4**
- **Figure S5**
- **Table S1.**
- **Supplemental experimental procedures**

Movie S1. Typical spontaneously contracting hESC-cardiomyocytes on micropatterned polyacrylamide in control medium or TID-containing medium. Related to Figure 4.

Movie S2. Typical spontaneously contracting hiPSC-cardiomyocytes, control or HCM, on micropatterned polyacrylamide in TID-containing medium. Related to Figure 5.

Supplementary figure 1

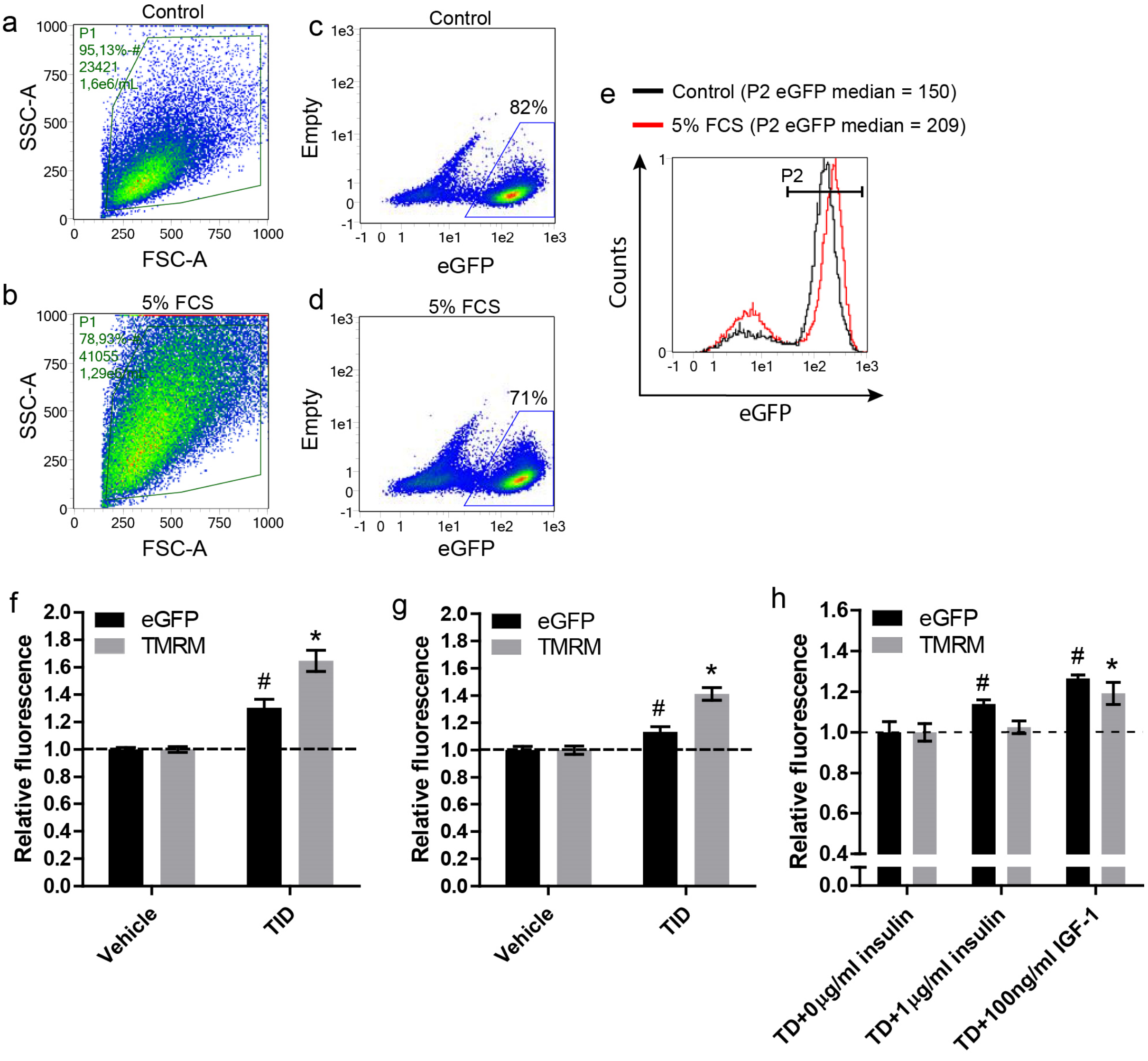

Figure S1. GFP and TMRM measurements in hPSC-derived cardiomyocytes maintained in different conditions. Related to Figure 1. FACS measurement after cardiac differentiation of *NKX2-5*$^{eGFP/w}$ hESCs and maintenance for 5 days under control conditions or in the presence of 5% FCS. (**a**) Scatter plots and P1 gating of control and (**b**) 5% FCS treated cells, (**c**) eGFP fluorescence of the P1 population of control and (**d**) FCS treated cells, (**e**) eGFP fluorescence histogram showing an overlay of the two P1 populations. The median eGFP fluorescence value within the P2 gate is shown above the histogram. (**f**) Relative TMRM and eGFP fluorescence values of M1 *NKX2-5*$^{eGFP/w}$ hESC-derived cardiomyocytes and (**g**) *NKX2-5*$^{eGFP/w}$ hiPSC-derived cardiomyocytes after 5 days of treatment with vehicle-only or TID medium. (**h**) Relative TMRM and eGFP fluorescence of cardiomyocytes after 5 days of treatment with T3+Dex (TD) plus insulin or IGF-1. Data are mean±SEM, *n*=6 (f), *n*=8 (g), *n*=9 (h). The *n* signifies the number of wells (cell populations) measured. Statistical significance was calculated using a one-way ANOVA with Dunnett's correction, # P<0.05 for eGFP fluorescence, * P<0.05 for TMRM fluorescence.

Supplementary figure 2

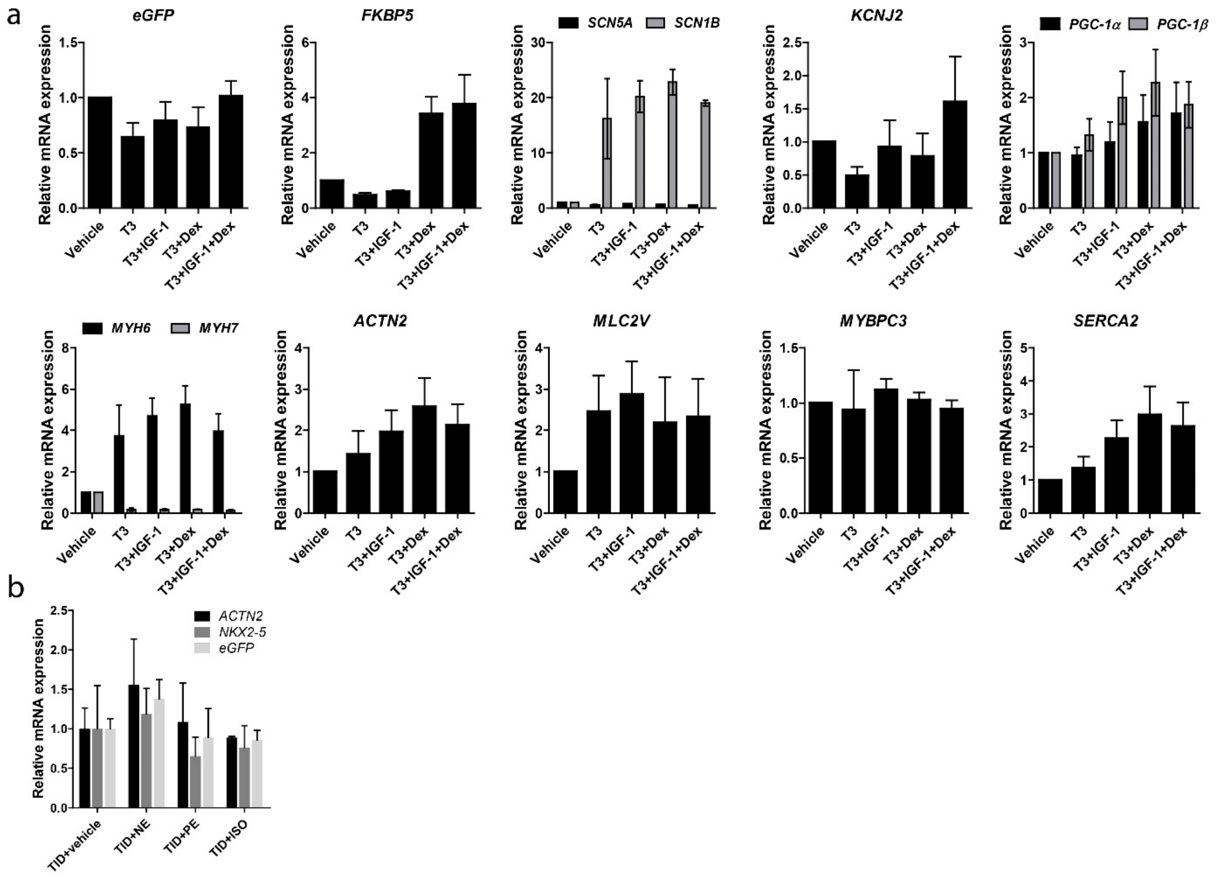

Figure S2. Gene expression profiles in hESC-derived cardiomyocytes maintained in different conditions. Related to Figure 1. (**a**) hESC-derived *NKX2-5*$^{eGFP+}$ cardiomyocytes were isolated by FACS after 7 days of treatment with the conditions shown. Gene expression is normalized to the housekeeping gene *RPLP0* and shown relative to the vehicle-only treated control. (**b**) Gene expression in cardiomyocytes treated for 5 days with T3+IGF-1+Dex (TID) plus vehicle, 1 µM norepinephrine (NE), 10 µM phenylephrine (PE) or 1 µM isoproterenol (ISO). Gene expression is normalized to the housekeeping gene *RPLP0* and shown relative to the TID+vehicle treated control. Data are mean±SEM from three independent experiments in **a**, and mean±SD for 3 replicate wells in **b**.

Supplementary figure 3

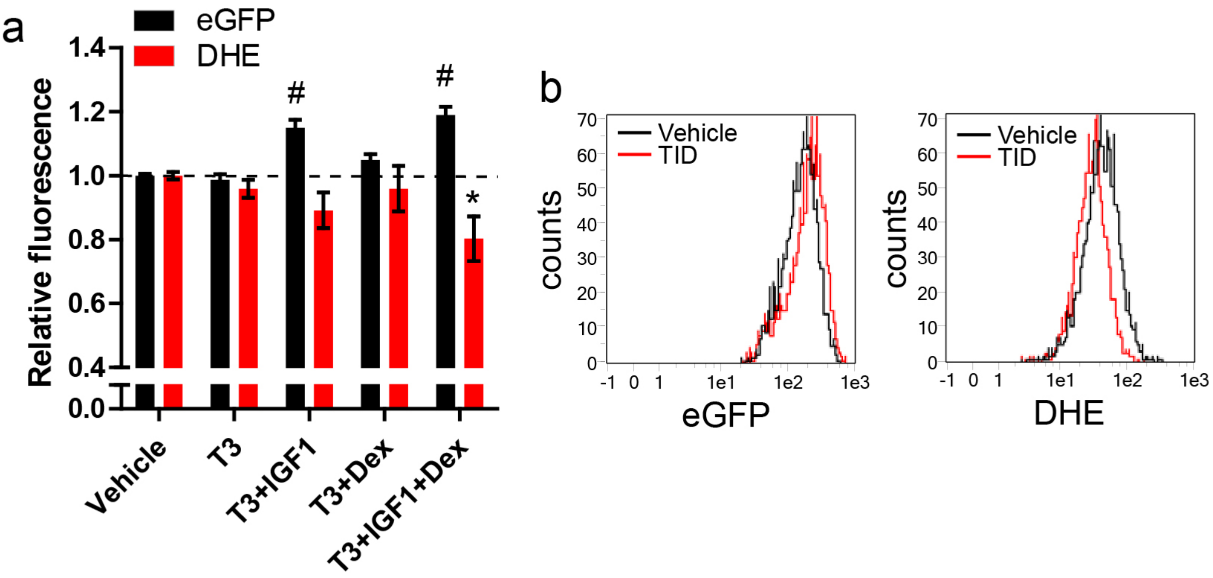

Figure S3. Modulation of reactive oxygen species levels in hESC-derived cardiomyocytes. Related to Figure 2. (**a**) Dihydroethidium (DHE) fluorescence measurements reporting superoxide radical levels in eGFP$^+$ cardiomyocytes shown relative to a vehicle-only control after 5 days of maintenance with the factors shown. (**b**) Histograms of eGFP and DHE fluorescence intensity in eGFP$^+$ cells maintained with vehicle-only or T3+IGF-1+Dex (TID). Note that eGFP is slightly increased while DHE is decreased by TID. Data are mean±SEM from three independent experiments. Statistical significance was calculated using a one-way ANOVA with Dunnett's correction, # $P<0.05$ for eGFP fluorescence, * $P<0.05$ for DHE fluorescence.

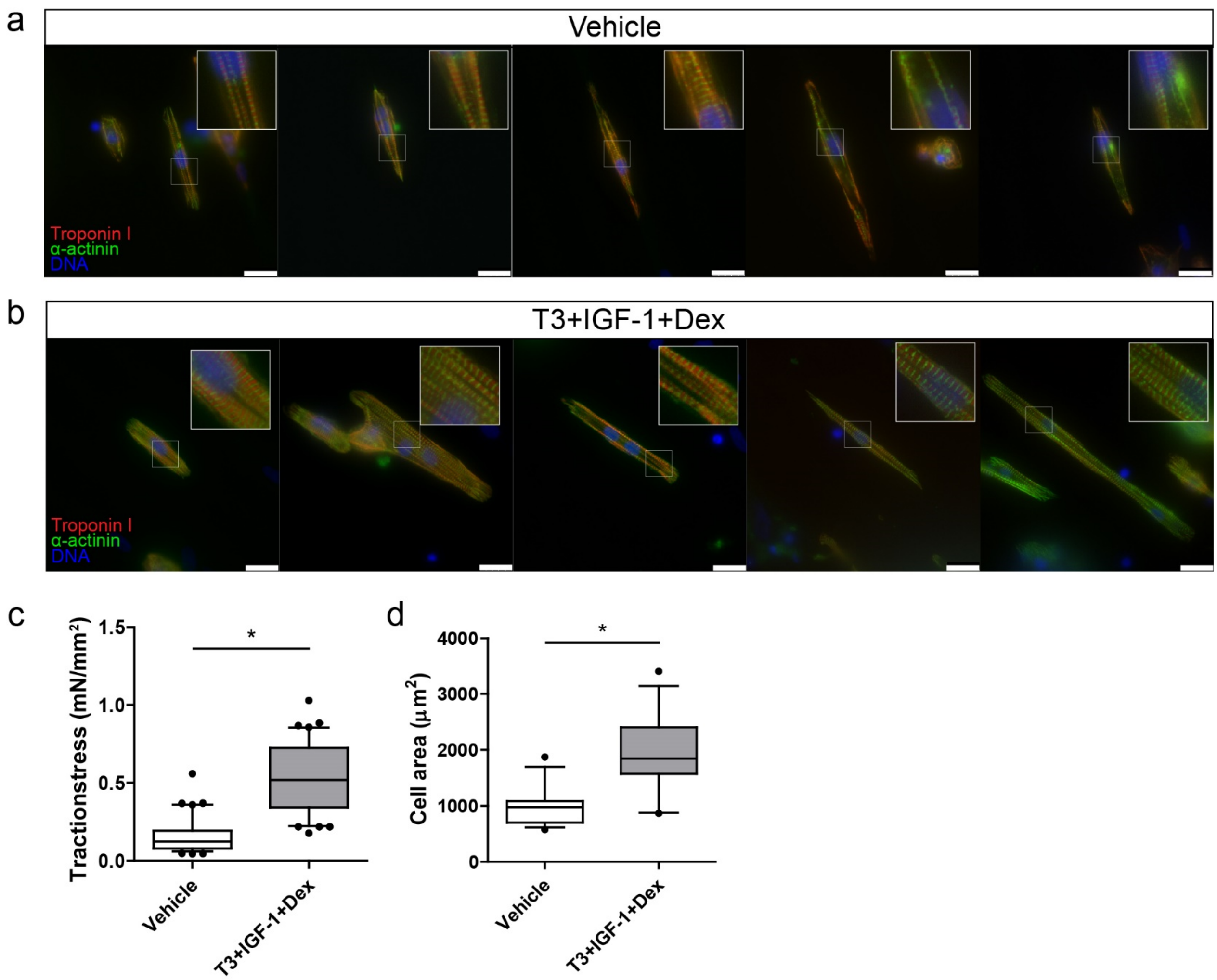

Figure S4. Structural imaging of hESC-derived cardiomyocytes and traction force analysis in an additional cell line. Related to Figure 4. (**a**) Immunostaining of Troponin I and α-actinin in cardiomyocytes maintained in (**a**) vehicle-only and (**b**) TID-medium, aligned on micropatterned PDMS gels. (**c**) Traction stress and (**d**) cell area of M1 hESC-derived cardiomyocytes maintained in vehicle or TID medium (*n*=15 individual cell measurements for each condition). Box and whisker plots show the median, interquartile range and 10-90 percentile range. Statistical significance was calculated with an unpaired t-test, * $P<0.05$.

Supplementary figure 5

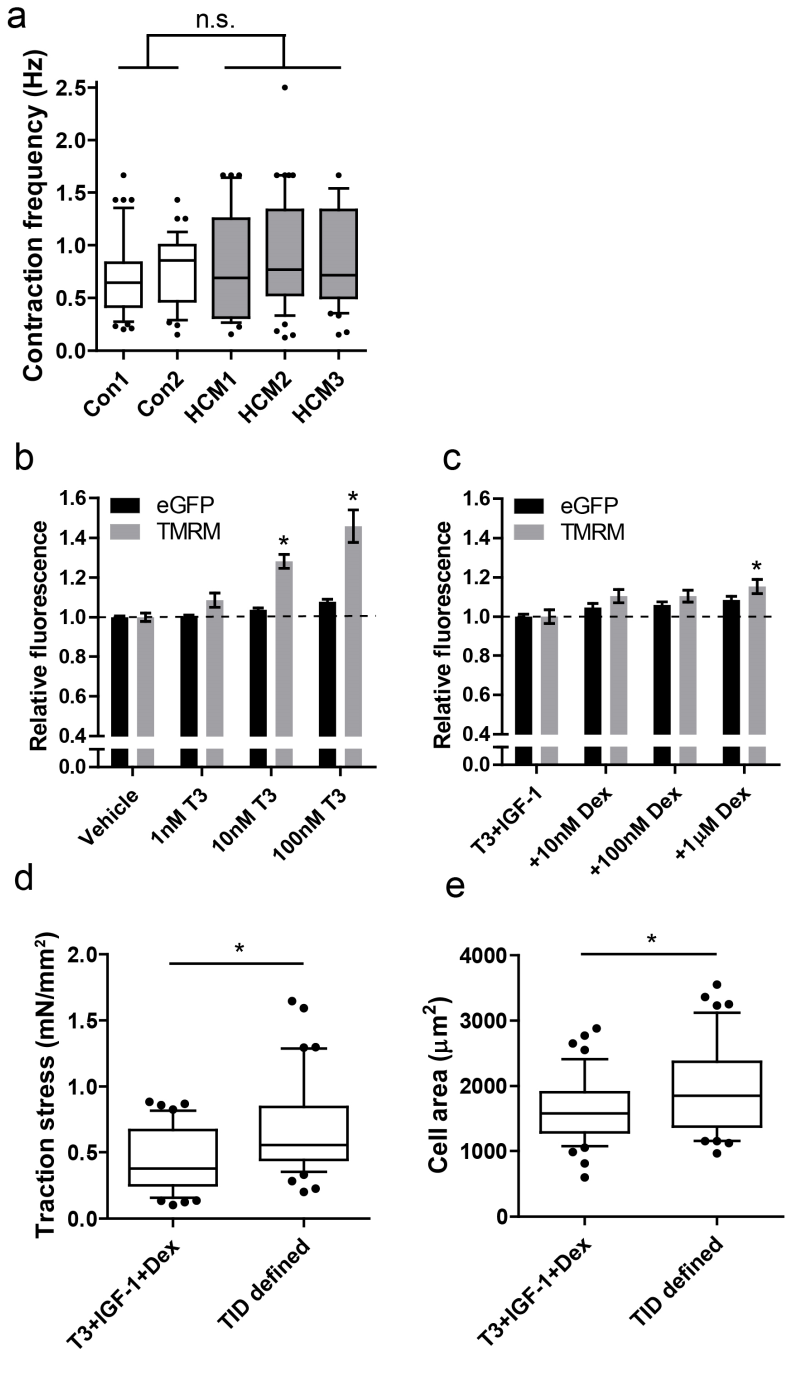

Figure S5. HCM cardiomyocyte frequency analysis and testing TID in a defined, albumin-free, basal medium. Related to Figure 5. (a) Contraction frequency of single spontaneously contracting iPSC-derived cardiomyocytes from two control (Con1 [*n*=47] and Con2 [*n*=36]) and three *MYBPC3* mutation lines (HCM1 [*n*=44], HCM2 [*n*=54] and HCM3 [*n*=43]). Box and whisker plots show the median, interquartile range and 10-90 percentile range. (**b**) eGFP and TMRM fluorescence values in eGFP$^+$ cardiomyocytes relative to a vehicle-only control after 5 days of incubation with the concentrations of T3 shown (*n*=6). (**b**) eGFP and TMRM fluorescence values in eGFP$^+$ cardiomyocytes maintained in T3

(100 nM) +IGF-1 supplemented with a range of dexamethasone concentrations, measured relative to the T3+IGF-1+vehicle control (T3+IGF-1). (**d**) Traction stress and (**e**) cell size of single spontaneously contracting cardiomyocytes maintained in T3+IGF-1+Dex (*n*=45) and T3+IGF-1+Dex in defined medium (*n*=43). The *n* signifies independent experimental replicates in **b** and **c** and the number of individual cells in **a**, **d** and **e** acquired over three independent experiments. Statistical significance was calculated with a one-way ANOVA with Tukey's multiple comparison test in **a**, a one-way ANOVA with Dunnett's correction in **b** and **c**, and an unpaired t-test in **d** and **e**. n.s. = not significant, * P<0.05.

Table S1. Clinical Data of HCM patients with 2373insG mutation in *MYBPC3.* Related to Figure 5.

Patient	Age	Sex	Interventricular Septum in Diastole, mm	LVEDD, mm	LVESD, mm	% FS	LVEF%
HCM1	44	m	13	54	32.9	39	56
HCM2	14	m	35	41	24	41	58
HCM3	42	m	24	55	42	24	48
Healthy Range			<13	36-56	20-40	25-43	55-70

LVEDD = left ventricular end diastolic diameter; LVESD = left ventricular end systolic diameter; % FS = fraction shortening (LVEDD-LVESD)/LVEDD*100%; LVEF% = left ventricular ejection

Supplemental Experimental Procedures (refers to Experimental Procedures)

hPSC culture and differentiation

H3 *NKX2-5*$^{eGFP/w}$ hESCs or M1 NKX2-5eGFP/w hESCs as previously generated (Elliott et al., 2011), were maintained on mouse embryonic fibroblasts and passaged using TrypLE select (Life Technologies). The generation of transgene-free hiPSCs from skin fibroblasts of one healthy male donor (LUMC0004iCtrl [Con1]) and three patients each with a c.2373dupG mutation in *MYBPC3* (LUMC0033iMyBPC [HCM1], LUMC0034iMyBPC [HCM2] and LUMC0035iMyBPC [HCM3]) was previously reported (Dambrot et al., 2014). A second transgene-free control hiPSC line (LUMC0047iCtrl [Con2]) generated from another healthy male donor was included in this study. hiPSCs were maintained on Matrigel (growth factor reduced; Corning 354230) in mTeSR1 medium (Stem Cell Technologies) and passaged with 1 mg/ml Dispase (Life Technologies).

Cardiac differentiation was induced from monolayer cultures on Matrigel in serum-free medium (BSA, polyvinyl alcohol, essential lipids [BPEL]) as previously described (Ng et al., 2008). The formulation was as follows: 44% IMDM, 44% Ham's F12, 5% protein-free hybridoma medium (PFHM-II), 0.25% BovoStar BSA (Bovogen, Australia), 0.125% polyvinyl alcohol, 1x chemically defined lipids, 400 µM α-thioglycerol (Sigma-Aldrich), 50 µg/ml L-ascorbic acid 2-phosphate (AA-2P), 2mM Glutamax, 0.1x Insulin-transferrin-selenium-ethanolamine (ITS-X) and 0.5% Pen/Strep. All BPEL components were obtained from Life Technologies unless otherwise stated. The following factors were present for the first 3 days 20 ng/ml BMP4 (R&D), 20 ng/ml Activin A (Miltenyi Biotech) and 1.5 µM CHIR 99021 (Axon Medchem). 5 µM XAV 939 (Tocris Bioscience) was present on days 3-6. *NKX2-5*$^{eGFP/w}$ hiPSCs (RD, CLM unpublished) were maintained in Essential 8 medium (Life Technologies) and differentiated as previously described (van den Berg 2015).

Contracting cultures were dissociated on day 13 and replated on Matrigel-coated 24-well plates. The following experimental factors were added on day 16, refreshed on day 20 and measured on day 21: 100 ng/ml Long R3 IGF-1 (in the main text: IGF-1), 1 µM SAG (Millipore), 1 µM dexamethasone, 100 nM triiodothyronine hormone, 10 µM phenylephrine, 1 µM isoproterenol, 1-1000 nM norepinephrine. The above factors were obtained from Sigma Aldrich unless otherwise stated.

The defined cardiomyocyte medium used in the *MYBPC3* shRNA experiment was composed of the following: DMEM (Sigma D5030), 15 mM glucose, 0.5 mM sodium pyruvate, 0.19 mM sodium hydroxybutyrate, 0.5 mM L-carnitine, 1 mM creatine, 5 mM taurine, phenol red (0.011 g/l), 1x Trace elements (A, B and C; Corning), 1x chemically defined lipids (Life Technologies), 2 mM Glutamax, 400 µM α-thioglycerol, 0.1x ITS-X, 50 µg/ml AA-2P, 0.5% Pen-Strep, 3.5 g/l sodium bicarbonate, 100 nM T3, 100 ng/ml Long R3 IGF-1 and 1 µM dexamethasone.

Lentiviral transduction

shRNAs against *MYBPC3* (NM_000256) were obtained from Open Biosystems in the pLKO Puro vector (#1 - TRCN0000082906 (AGCCAGAATATGGTTGGCTTT) and #2 - TRCN0000082903 (CCTCCCTACTGTTGGATGTAT). A scrambled shRNA was used as control (Sarbassov et al., 2005) (Addgene plasmid: 1864). Cardiomyocytes were transduced with lentiviruses on day 15 of differentiation and subsequently selected with puromycin for 4 days. Cultures were maintained for an additional 7 days before single cell dissociation onto the gelatin-patterned acrylamide gels for measurement 3 days later. Protein knockdown was assessed by Western blot.

Patterned Polyacrylamide gel fabrication

Patterned polyacrylamide gels were prepared as previously described (Rape et al., 2011). Briefly, a 1% gelatin solution was activated with 3.5 mg/mL Sodium Periodate (both Sigma-Aldrich). A polydimethylsiloxane (PDMS) stamp was casted from a SU8 master produced by standard soft lithography techniques and incubated with the activated 1% gelatin solution for 45 mins. The excess of gelatin was removed with a nitrogen gun and the stamp was used to µcontact-print a pattern of 20 µm thick with 20 µm spacing gelatin lines onto 10mm (electrophysiology) or 15 mm (contraction assay) coverslips. The polyacrylamide solution was prepared with a final concentration of 0.1% bis-acrylamide (Bio-Rad), 5% acrylamide (Bio-Rad) and 10 mM HEPES pH 8.5 in distilled water, followed by centrifugation for 1 min at 10.000 RPM for degassing. 0.006% (m/v) of ammonium persulfate (Sigma-Aldrich) and a 1:1000 dilution of 0.2-µm fluorescent beads (Ex/Em: 660/680nm - Molecular Probes) were added to the solution (for the contraction assay) and briefly vortexed. The gel polymerization was initiated with TEMED (Bio-Rad) and 4.08 µl/9.2 µl of the final solution was added to a 15 mm/25 mm coverslip treated with plus Bind-Silane solution (GE Healthcare). The µcontact-printed coverslip was applied on top of the drop with the gelatin lines facing the gel. After 20mins of polymerization the 10 mm/15 mm coverslip was removed. Each 25mm coverslip was mounted onto a well of a glassbottom 6-well plate (Mattek), replacing the initial glass. The 15mm coverslips were used directly. Plates were UV-sterilized and re-hydrated with culture medium for 30 mins before use. The polymerized gel has a Young's modulus of 5.8 kPa (Frey et al., 2007).

Flow cytometry measurements for plasma membrane potential and reactive oxygen species

For relative plasma membrane potential measurement, differentiated cultures were dissociated on day 21 (after treatment as above from day 16) using 5x TrypLE Select and resuspended in 2.5 nM TMRM (Life Technologies) in warm assay medium. Cells were incubated for 8 minutes at 37°C before being measured by flow cytometry. eGFP and TMRM fluorescence intensities were recorded. For superoxide detection, dissociated cells were labelled with 5 µM DHE for 30 mins at 37°C and then measured by flow cytometry. Samples were measured with a MACSQuant VYB (Miltenyi Biotech)

equipped with a blue (488nm) and yellow (561nm) laser. eGFP and DHE fluorescence intensities were recorded. Appropriate compensation to correct from cross-bleed was performed for each.

Respiration and acidification rates measured with the Seahorse XF-24 Analyzer

Respiration and acidification rates were measured on adherent cells using a Seahorse XF-24 or an XF-96 Analyzer (Seahorse Bioscience). The assay plates were coated with Matrigel (1:100). Cells were seeded in BPEL medium at a density of $6.5x10^4$ (XF-24) or $2.5x10^4$ (XF-96) cells/well 7 days before measurement. The assay was performed in bicarbonate-free DMEM (D5030; Sigma Aldrich), containing 0.25% fatty acid-free BovoStar (Bovogen, Australia), 1 mM sodium lactate, 0.15 mM sodium hydroxybutyrate, 2 mM GlutaMAX (Life Technologies) and 0.5 mM L-carnitine. The standard glucose concentration was 15 mM glucose and was supplemented with 0.5 mM sodium pyruvate. Cells were washed twice and pre-incubated in the assay medium for 1 h before measurement. For the standard profiling oligomycin was used at 0.5 µg/ml, FCCP titrated in 2 injections to 3 µM and rotenone and antimycin A were added at 1 µM and 2 µM respectively. A standard protein assay was used to normalize values to whole cell protein. Acid titration experiments were performed to calculate the buffering capacity of the Seahorse assay media and gave a value of 0.1 pmoles H+/mpH in the 7 µl measuring volume. ECAR values were converted to anaerobic glycolyis/lactate production rates as previously described (Mookerjee et al., 2015), using a max H+/O2 value of 1.0 and converted 1:1 to an ATP production rate. The oligomycin-sensitive oxygen consumption was converted to an ATP production rate using a P/O ratio of 2.3 (Brand, 2005). To assess ATP demand for excitation and contractility nifedipine (10 µM) and blebbistatin (5 µg/ml) were co-injected and the respiration rate immediately recorded, followed by measurements after oligomycin and then rotenone and antimycin A injection. A vehicle-only injection was performed in parallel and the effect subtracted. Experiments analysing the effect of etomoxir and UK5099 were performed on the XF-96 format using an assay medium with the following modifications: the glucose concentration was 5.5 mM, sodium pyruvate 0.15 mM and palmitic acid was included at 100 µM conjugated to fatty acid-free BovoStar BSA in a 2.8:1 molar ratio. To prepare this a 4 mM palmitic acid solution was made in assay medium by heating at 65°C for 30 mins, then combined 1:1 with a 10% Bovostar BSA solution and heated at 37°C for 30 mins with mixing. 10 mM HEPES was also included in this assay medium to provide extra buffering (glycolytic rates were not calculated). Cells were washed twice and pre-incubated in this assay medium for 4 h before measurement. To assess the response to substrate uptake inhibitors, 40 µM etomoxir or 5 µM UK5099 were injected and respiration rates recorded after 45 mins, followed by injections of oligomycin, FCCP (3 µM) and then rotenone and antimycin A. A vehicle-only injection was performed in parallel and the effect subtracted.

Electrophysiological characterization

Action potential (AP) recordings were performed on single cardiomyocytes, 14-16 days after cell dissociation with the amphotericin perforated patch-clamp technique using an Axopatch 200B amplifier (Molecular Devices Corporation, Sunnyvale, CA, USA). Signals were filtered and digitized at 5 and 40 kHz, respectively. Data acquisition and analysis were accomplished using pClamp10.1 (Axon Instruments) and custom software. Potentials were corrected for the liquid junction potential. Cells were continuously perfused in a perfusion chamber at 37 ^{0}C (Cell MicroControls Norfolk VA, U.S.A.) using Tyrode's solution containing (mM): NaCl 140, KCl 5.4, CaCl2 1.8, MgCl2 1.0, glucose 5.5, HEPES 5; pH 7.4 (NaOH). Pipettes (borosilicate glass; resistance ~2.5 MΩ) were filled with solution containing (mM): K-gluconate 125, KCl 20, NaCl 5, amphotericin-B 0.22, HEPES 10; pH 7.2 (KOH).

APs were recorded at spontaneous frequencies and characterized by duration at 50 and 90% repolarization (APD50, and APD90, respectively), maximal diastolic potential (MDP), AP amplitude, maximal upstroke velocity and frequency. AP parameter values obtained from 8-9 consecutive APs were averaged and data were collected from at least 2 independent differentiations per condition.

Traction force measurements

The traction force measurements were performed as previously described (Hazeltine et al., 2012). Cells were seeded on gelatine patterned acrylamide gels 4 days before and measured in their normal culture medium in an environment maintained at 37°C with 5% CO_2. Briefly, using a Leica AF-6000LX microscope, an image-series of aligned single spontaneously contracting cardiomyocytes was taken at 40x magnification at 20 frames per second, recording brightfield and fluorescent beads. Single frames from maximal relaxation and contraction of the brightfield and fluorescent beads image-series were analyzed by the LIBTRC software package (kindly provided by Dr. Micah Dembo), creating a mask of the cell outline from the brightfield image and a vector map from the difference between the relaxed and contracted fluorescent beads images. The vector map and the cell mask were used to calculate the maximum total force that the cell applies on the substrate at its peak of contraction. The traction stress generated by the cardiomyocyte during contraction was calculated by dividing the total force by the cell surface area. Measurements were blinded in acquisition and analysis.

Quantitative real-time PCR

RNA was isolated using a Minelute RNA extraction kit (Qiagen) and cDNA synthesized using an iScript cDNA synthesis kit (BioRad). Real-time PCR was performed on a BioRad CFX384 machine using IQ SYBR Green (BioRad). Gene expression values were normalized to the mean expression of the housekeeping genes human ribosomal protein (*RPLP0*), glucuronidase (*GUSB*), and *RNF7*. Primer sequences were as follows:

Gene	Forward primer	Reverse primer
RPLP0	CACCATTGAAATCCTGAGTGATGT	TGACCAGCCCAAAGGAGAAG
GUSB	CCACCTAGAATCTGCTGGCTAC	GTGCCCGTAGTCGTGATACCAA
RNF7	ACGCACCGAATAGTTACGG	CCAGGTCCACGGGCAGA
eGFP	GTGAGCAAGGGCGAGGAG	CCGTAGGTCAGGGTGGTC
FKBP5	TTCTCCTTGCTGCCTTTCTG	ACCCTTGGCTGACTCAAACTC
SCN5A	GAGCTCTGTCACGATTTGAGG	GAAGATGAGGCAGACGAGGA
SCN1B	GTGGTTGTAGGTGACATTGGTG	GAAGGGCACTGAGGAGTTTGT
KCNJ2	ACCGCTACAGCATCGTCTCT	TCCACACACGTGGTGAAGAT
PGC-1α	AACACTTACAAGCCAAACCA	GGGTTCAATAGTCTTGTTCTC
PGC-1β	TCCTCAACTATCTCGCTGAC	CTCACTGTCAATCTGGAAGAG
MYH6	CTTCTCCACCTTAGCCCTGG	GCTGGCCCTTCAACTACAGA
MYH7	CGACCTTCTTCTCTTGCTC	GAGGACAAGGTCAACACCCT
ACNT2	CTGCTGCTTTGGTGTCAGAG	TTCCTATGGGGTCATCCTTG
MYL2/MLC2V	TACGTTCGGGAAATGCTGAC	TTCTCCGTGGGTGATGATG
MYBPC3	GGCATGCTAAAGAGGCTCAA	TCTTGTGGCCTTTGCTCAC
SERCA2	ACCCACATTCGAGTTGGAAG	CCAACGAAGGTCAGATTGGT

Western blotting

The samples were lysed with ice cold ELB (50mM HEPES pH 7.0; 250mM NaCl; 5mM EDTA; 0.1% NP-40) with 1:100 Protease Inhibitor Cocktail (Sigma-Aldrich) for 30 mins on ice. The samples were centrifuged at 7,000g for 10 mins and the supernatant was quantified for protein content using the Bio-Rad Protein Assay (based on the Bradford dye-binding method). 100µg of the hiPSC-CM lysates were run in a 6% polyacrylamide gel and 30µg of the hESC-CM with the shRNA lysates were run in a 10% polyacrylamide gel together with the protein ladder (Precision Plus Protein dual colour – Bio-Rad). The protein was transferred to an Amersham Hybond membrane (GE Heathcare- Life Sciences) overnight. The membrane was blocked with blocking buffer (2% milk in PBS) and each protein was detected with the specific antibody: Rabbit IgG anti- cMyBP-C (1:2000) kindly provided by Dr. Sakthivel Sadayappan from the Department of Cell and Molecular Physiology, Health Sciences Division, Loyola University Chicago, followed by Horse anti-Rabbit IgG – HRP (1:2000) (Cell Signaling). Mouse IgG anti-Actin (1:1000) (MAB1501 – Millipore) and Mouse IgG anti-α-actinin (A7811-Sigma-Aldrich), followed by Goat anti-mouse IgG-HRP (1:2000) (Cell Signaling).

Supplemental references

Frey, M.T., Engler, A., Discher, D.E., Lee, J., and Wang, Y.-L. (2007). Microscopic methods for measuring the elasticity of gel substrates for cell culture: microspheres, microindenters, and atomic force microscopy. Methods Cell Biol. *83*, 47–65.

Rape, A.D., Guo, W.-H., and Wang, Y.-L. (2011). The regulation of traction force in relation to cell shape and focal adhesions. Biomaterials *32*, 2043–2051.
